# Prevalence and pathology of *Cephalopina titillator* infestation in *Camelus bactrianus* from Xinjiang, China

**DOI:** 10.1186/s12917-022-03464-5

**Published:** 2022-09-28

**Authors:** Huaibing Yao, Mengli Liu, Wanpeng Ma, Haitao Yue, Zhanqiang Su, Ruiqi Song, Qiang Ma, Ling Li, Zhuangyuan Wu, Yingjun Ma, Gangliang Chen, Baojiang Chen, Jie Yang

**Affiliations:** 1 College of Life Sciences and Technology, Xinjiang University, Urumqi, Xinjiang China; 2 Key Laboratory of Biological Resources and Genetic Engineering, Urumqi, Xinjiang China; 3 College of Health Management, Xinjiang Medical University, Urumqi, Xinjiang China; 4 College of Veterinary Medicine, Xinjiang Agricultural University, Urumqi, Xinjiang China; 5 College of Animal Science and Technology, Shihezi University, Shihezi, Xinjiang China; 6 Bureau of Animal Husbandry and Veterinary, Altai, Xinjiang China; 7 Bactrian Camel Academe of Xinjiang, Xinjiang Wangyuan Camel Milk Limited Company, Altai, Xinjiang China

**Keywords:** *Cephalopin atitillator*, Prevalence, Pathology, Life cycle, Molecular identification

## Abstract

**Background:**

In camels, nasopharyngeal myiasis is caused by the larvae of *Cephalopina titillator*, which parasitize the tissues of nasal and paranasal sinuses, pharynx, and larynx. *C. titillator* infestation adversely affects the health of camels and decreases milk and meat production and even death. However, the *C. titillator* infestation in Bactrian camels has not been widely studied.

**Methods:**

The present study was conducted to determine the prevalence and risk factors of *C. titillator* in Bactrian camels of northwestern Xinjiang. Suspected larvae recovered from infested camels were evaluated for *C. titillator* by microscopy and polymerase chain reaction. Nucleotide sequences of the partial mitochondrial cytochrome c oxidase subunit I (*COX1*) and cytochrome b (*CYTB*) genes from the *C. titillator* of camels were aligned from the NCBI database. Furthermore, the gross and histopathological alterations associated with *C. titillator* infestation were evaluated via pathological examination.

**Results:**

Of 1263 camels examined 685 (54.2%) camels were infested with suspected *C. titillator* larvae. Different larval stages were topically detected in the nasal passages and pharynx of the camel heads. Microscopy analysis of the pharyngeal mucosa tissue revealed necrotic tissue debris and some inflammatory cells. Molecular detection of the larval *COX1* and *CYTB* genes indicated that pathogen collected in Bactrian camels was *C. titillator*. The epidemiological study demonstrated that the prevalence rate of *C.titillator* infestation was significantly higher in camels of Bestierek Town Pasture (67.2%) and Karamagai Town Pasture (63.6%) compared to Kitagel Town Pasture (38.7%) and Qibal Town Pasture (35.8%) (*P* < 0.05). No significant difference was observed between the prevalence rates in male (52.6%) and female (54.6%) camels (*P* > 0.05). The prevalence was higher in warm (64.2%) than that in cold (48.4%) seasons (*P* < 0.001). The prevalence in camels with non-nomadic method (67.2%) was significantly higher than in animals with nomadic method (47.5%) (*P* < 0.001). The prevalence of *C.titillator* infestation was significantly higher in animals of aged 5–10 (60.1%) and aged > 10 (61.1%) years old compared to those of aged < 5 (31.7%) years old camels (*P* < 0.001).

**Conclusion:**

Our results confirm that there is a high prevalence of *C. titillator* in Bactrian camels from Xinjiang, closely related to age, season, pasture environment, and husbandry methods. Developing prevention, diagnosis, and control programs to prevent transmission is necessary.

**Supplementary Information:**

The online version contains supplementary material available at 10.1186/s12917-022-03464-5.

## Key findings

- Epidemiological analyses of *Cephalopina titillator* infestation in *Camelus bactrianus* in Xinjiang were performed.
- Camel nasopharyngeal myiasis appears as fly strike and myiasis.
- *C. titillator* life cycle and the clinicopathological changes in camels were reported.
- Morphological and molecular analyses were performed to examine *C. titillator* characteristics in Bactrian and dromedary camels.

## Background

*Cephalopina titillator* is considered a Palaearctic species that has extended its range into camel-rearing areas in the world, following the domesticated host [1]. Nasopharyngeal myiasis in camels is caused by the larvae of *C.titillator* (Clark,1797), an obligate parasite that infects only camelids and of belongs to the Oestridae family [2, 3]. *C. titillator* larvae infestation adversely affects animal health, suppresses host physiological and immunity functions, damages host tissues, and causes severe economic losses to the camel breeding industry [4, 5] by decreasing milk and meat production and fertility. This is a high incidence parasitic disease among all breeds of camels in the Middle East, Africa, and Asia [6]. The prevalence of *C. titillator* infestation in Saudi Arabia, Nigeria, Egypt, Jordon, Libya, Iraq, Iran, and Inner Mongolia of China is reported to be 91%, 58%, 37%, 33%, 79%, 42%, 52.3%, and 42.5%, respectively [2, 6–13]. Recent studies have reported *C. titillator* infestations in llamas and alpacas [14]. Female flies deposit their eggs into the nasal cavity of the camels. Subsequently, the hatched larvae crawl into the nasopharynx and sometimes the paranasal sinuses without markedly affecting the nasal mucosa but elicit inflammation and promote tissue damage [15]. The severity of clinical symptoms depends mainly on the number of larvae and the damage to the host tissue during migration [16, 17]. Earlier studies have examined the infestation rates of *C. titillator* in dromedary camels from some countries in the Middle East [18–20]*.* However, the limited information is available on the epidemiological characteristics of parasitic infestations [12], especially myiasis, in Bactrian camels owing to their geographical distribution and late understanding of economic application value [21]. Some parasites have been reported to affect health and productivity of Bactrian camels, including the presence of intestinal parasites and ectoparasite (e.g., *Giardia duodenalis* [22], *Nematodirus SP*. [23, 24], *Chabertia* [24], *Haemonchus contortus* [24], *Coccidia* [24], *Ticks* [25]). Only simple cases of two-humped camel nasal myiasis have been reported in the early Chinese literature from the Inner Mongolia area. Moreover, the detection of high infestation rate and occurrence of *C. titillator* is challenging using the currently available immunodiagnostic technology. *C. titillator* infestation is difficult to detect in live camels, and it is also difficult to differentiate between other neurological or respiratory pathogens, and infestation with *C. titillator* larvae which has the same clinical symptoms [26]. The specific purified protein fraction extracted from the *C. titillator* larvae for the sensitive and specific diagnosis of early infestation in living camels [27]. Some researchers have performed indirect Enzyme Linked Immunosorbent Assay (ELISA) to diagnose in camel serum using purified fractions as antigens [28–30]. Currently, there are no diagnostic kits commercially available for detecting myiasis in camels. Mitochondrial DNA is considered a powerful genetic marker in the investigations and studying of the evolution and taxonomy of animal populations. A recent parasitological study of camels reported mitochondrial genomes of *C. titillator* (camel nasal bot fly), *Cephenemyia trompe* (reindeer throat bot fly), and *Rhinoestrususbekistanicus* (equine nasal bot fly) [31]. Previous research reports that partial COI and 16S ribosomal RNA (16S rRNA) genes sequencing can be used to identify the *C. titillator* of Camels [32, 33].

Therefore, this study aimed to identify and characterize *C. titillator* species with morphology and molecular genetics, and to describe the life cycle of the *C. titillator* and histopathological changes in the throat of infested camels. An epidemiological survey was conducted to clarify the prevalence and risk factors associated with *C. titillator* in camels.

## Results

### Gross pathology

**Fig. 1:**
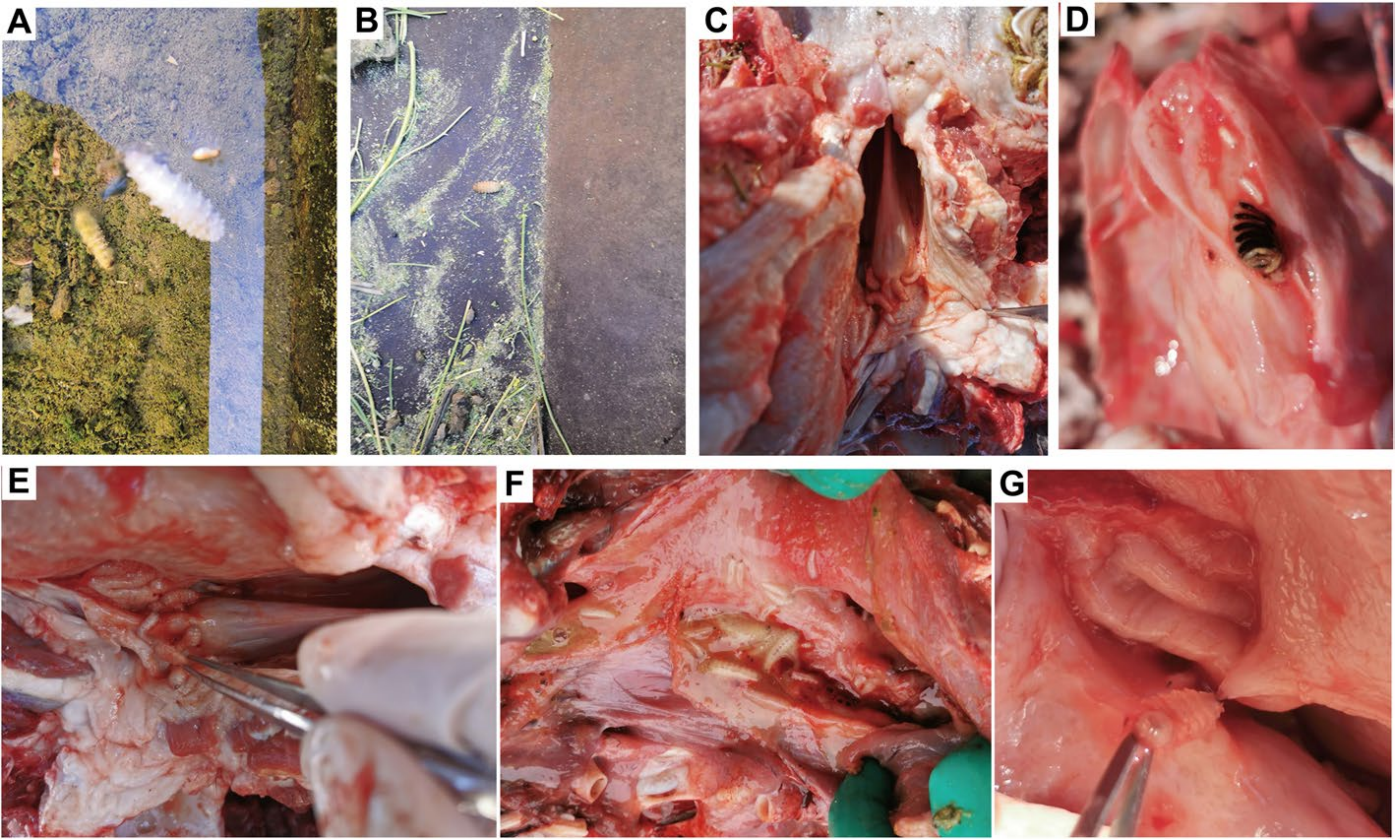
The collection of the *C. titillator*. **A-B** The expelled larvae were found in the drinking fountain and feed trough; **C-G** The heads of slaughtered camel showing the presence of three stages of *C. titillator* larvae in the pharynx; **C; E** The detected third-stage larvae; **D** The detected first-stage larvae; **F** The detected first-stage and second-stage larvae; **G** A larva still adhered firmly to the nasopharyngeal mucosa with their two sharp black frontal hooks under pulled by forceps tip

The infested camels sprayed third-stage larvae that were detected in the stables, especially in the drinking fountains (Fig. 1A) and feed troughs (Fig. 1B). Different larval stages were topically detected in the nasal passages and pharynx of the infested camel heads (Fig. 1C–G). The *C. titillator* larvae in the nasopharynx were alive and exhibited squirming and crawling even after several days post-slaughtering of the infested camels. Additionally, the *C. titillator* larvae anchored firmly to the mucous membrane through its sharp black mouth hooks (Fig. 1G). Most larvae were attached to the mucous membrane of the nasopharynx, whereas some larvae were found in the nasal cavity. Moderate numbers of *C. titillator* larvae within the nasal and paranasal sinuses do not cause damage. However, severe infestations cause irritation, tissue damage, respiratory disorders, and even death.

### Histopathology

**Fig. 2:**
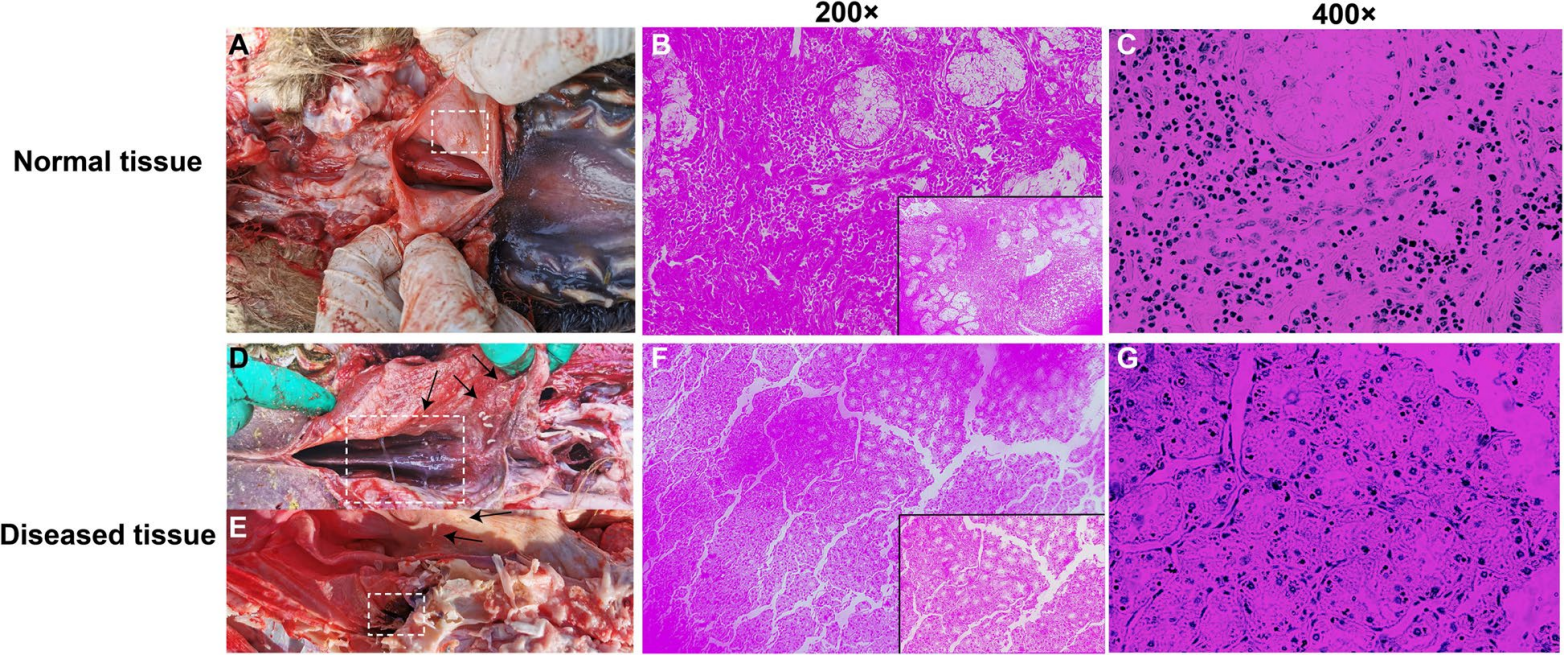
Histopathological examination. Nasopharyngeal region of *C. titillator* infested camel showing heamorrhagic, swollen, edematous and necrotic mucosa. **A** Heathly laryngeal and pharyngeal mucosa of non-infested camel with *C. titillator*; **B-C** Heathly laryngeal and pharyngeal mucosa (H&E × 200 and × 400); **D-E** The mucous membrane of the nasopharynx was swollen, edematous, occasionally liquefaction necrosis; **F-G** Nasopharyngeal region of *C. titillator* infested severely camel showing necrosis, fragmentation, and dissolution of laryngeal and pharyngeal mucosa cells (H&E × 200 and × 400)

Histopathological examination of the infested camel head revealed tissue damage mainly in the pharyngeal, nasopharyngeal cavity, and turbinate tissues, which are regions to which the larvae attached. Microscopy analysis of the pharyngeal mucosa tissue revealed necrotic tissue debris and some inflammatory cells. The laryngeal and pharyngeal mucosae of the un-infested camel were healthy (Fig. 2A–C)*.* In contrast, the nasopharyngeal mucosal membrane of the infested camel was swollen, edematous, occasionally associated with liquefaction necrosis, and comprised large amounts of dark-colored inflammatory exudates (Fig. 2D–E).In some severe cases, ulcer-like injuries and dark brown or black nodules containing pus were observed in the mucous membrane, representing the lesions to which the larvae attached previously (Fig. 2E).Degenerated larvae and dead mature larval bodies were embedded between the turbinate bones and ethmoid area in some cases. The injury area in the affected region depended mainly on the level of and the number of larvae. Severe *C. titillator* infestations were associated with necrosis, fragmentation, and dissolution of laryngeal and pharyngeal mucosal cells (Fig. 2F–G). In some cases, pus-filled nodules were also observed.

### Morphological identification of the C. titillator life cycle stages

**Fig. 3:**
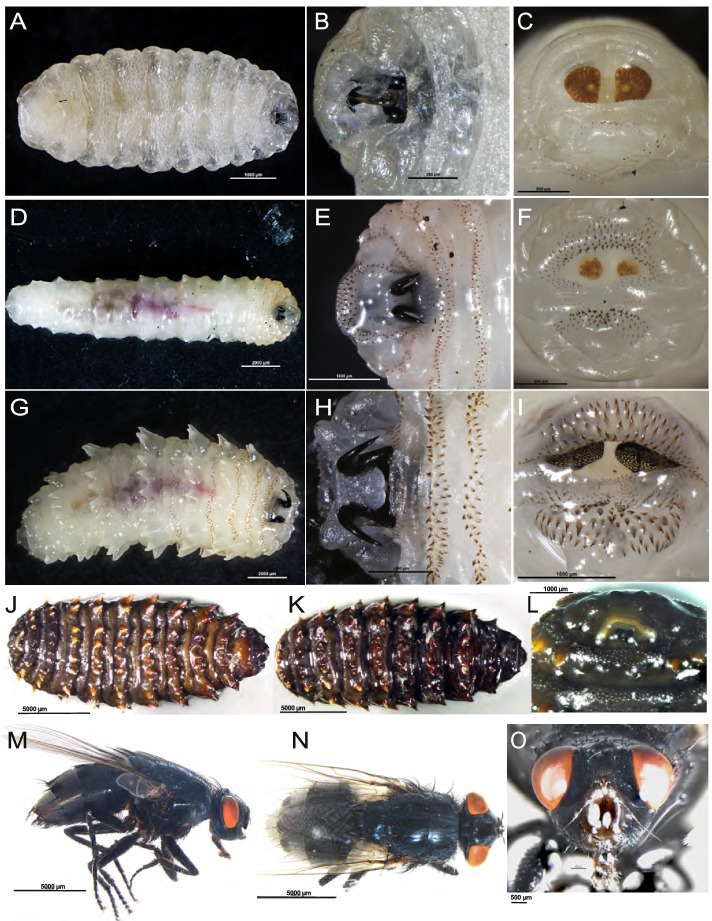
Photomicrographs of the different stages of *C. titillator*. **A** Ventral view of a first-stage larvae recovered from a camel (bar = 1000 μm) (L1); **B** Electron micrograph of the cephalic segment of a first-stage larvae (bar = 250 μm); **C** Electron micrograph of the terminal abdominal segment of a first-stage larvae (bar = 500 μm); **D** Ventral view of a second-stage larvae recovered from a camel (bar = 2000 μm) (L2); **E** Electron micrograph of the cephalic segment of a second-stage larvae (bar = 1000 μm); **F** Electronic micrograph of the terminal abdominal segment of a second-stage larvae (bar = 500 μm); **G** Ventral view of a third-stage larvae recovered from a camel (bar = 2000 μm) (L3); **H** Electron micrograph of the cephalic segment of a third-stage larvae (bar = 1000 μm); **I** Electron micrograph of the terminal abdominal segment of a third-stage larvae (bar = 1000 μm); **J** Electron micrograph of the flat abdomen of pupal stage of *C. titillator* (bar = 5000 μm) (Pu); **K** Electron micrograph of the raised back of pupal stage of *C. titillator* (bar = 5000 μm); **L** Electron micrograph of the mouthparts of pupal stage of *C. titillator* (bar = 1000 μm); **M** Lateral view of adult fly *C. titillator* (bar = 5000 μm) (Ad); **N** Dorsal view of adult fly *C. titillator* (bar = 5000 μm); **O** En face view of adult fly *C. titillator* (bar = 500 μm)

*C. titillator* resembles *Oestrus ovis* or a honeybee. The fly was stout with a grayish-brown color and a length of approximately 1 cm and covered with tiny hairs. The mouthparts were vestigial. The color of the freshly recovered first-stage and second-stage larvae from the throat was whitish. The first-stage larvae were small (length, 3.9 ± 1.2 mm; width, 2.0 ± 0.4 mm).The body comprised clusters of small spines and exhibited fusiform and segmented patterns (Fig. 3A). The cephalic segment was well-defined with prominent sensillae and mouth hooks (Fig. 3B). The larvae comprised a terminal sac that aids migration to the host and subsequently to an appropriate point of entry (Fig. 3C). The body length of the second-stage larvae was in the range of 1.1 ± 0.2 cm, whereas the maximum body width was in the range of 0.4 ± 0.1 cm. The ventral surface of the second-stage larvae had dark brown lines with all segments comprising several tubercles (Fig. 3D). The color of the third-stage larvae was whitish to yellowish when completely mature with a dark brown line on their ventral surface. The length and maximum width of the larvae were in the range of 2.3 ± 0.2 cm and 0.8 ± 0.3 cm, respectively (Fig. 3G). The aspect ratio of length to width was approximately 3:1. A pair of black mouth hooks was observed at the front of larvae (Fig. 3B, E, and H). Two spherical rear valves were observed at the center of the rear end (Fig. 3C, F, and I). The rear and lower black sharp mouth hooks were inverted eight-shaped and curved outwards. Back spiracles were observed at the posterior end of the twelfth segment in third-stage larvae (Fig. 3C, F, and I). The cuticle of third-stage larvae gradually turns blackish. The pupal cuticle of *C. titillator* was dark brown with a length of approximately 15.6 ± 1.6 mm (Fig. 3J–L). Flies of the family Oestridae are large robust flies. Adult flies of *C. titillator* emerged from a circular opening. The adult flies had rudimentary and functional oral mouthparts for feeding (Fig. 3M–O). Characteristic and diagnostic dorsal protuberances were observed on the adult abdomen. Adult female flies deposit larvae into the nostrils of camels. The second-stage and third-stage larvae were imaged using a scanning electron microscope for the morphological description of the surface ultrastructure.

### Morphological analyses by scanning electron microscopy

**Fig. 4:**
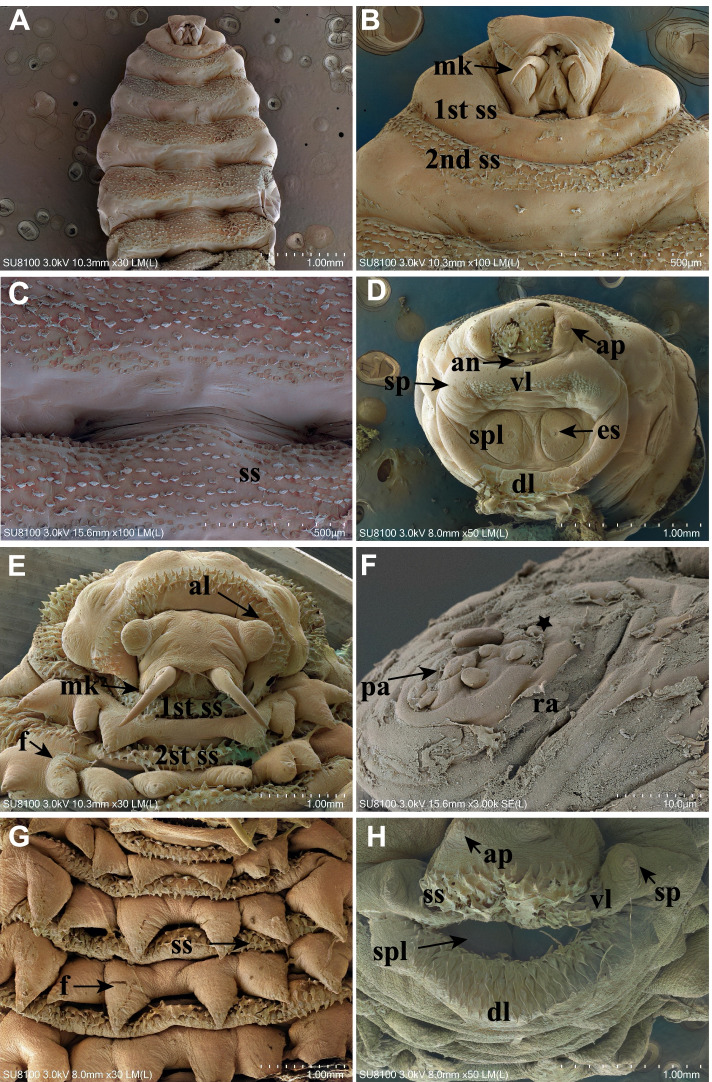
Scanning electron micrographs of *C. titillator*. **A** SEM of the cephalic, thoracic and abdominal segments of a first-stage *C. titillator* larvae (bar = 1.0 mm); **B** Ventral view of the cephalic and thoracic segments of first-stage *C. titillator* larvae (bar = 500 μm); **C** The small spines were numerous and irregularly distributed in dorsal and ventral rows in first-stage *C. titillator* larvae (bar = 200 μm); **D** SEM of the terminal abdominal segments of a first-stage *C. titillator* larvae (bar = 1.0 mm); **E** SEM of the ventral surface of the cephalic and thoracic segments of a third-stage *C. titillator* larvae recovered from a camel. Note the bases of antennal lobes are widely separated. Note the large number of spines (bar = 1.0 mm); **F** The third-stage *C. titillator* larvae papillae note the button-shaped papillae (bar = 10 μm); **G** SEM of the surface of the fleshy spines of a third-stage larvae (bar = 1.0 mm); **H **SEM of the terminal abdominal segment of a third-stage *C. titillator* larvae (bar = 1.0 mm)

As shown in Fig. 4, the small spinules (ss) were numerous and irregularly distributed in dorsal and ventral rows in second-stage larvae (Fig. 4A and C). The anterior end or pseudocephalon had long curved frontal mouth hooks (mk) (Fig. 4A–B and E). pseudocephalon with the first and second thoracic segments was supported by small spines (ss) (Fig. 4B and E).The last abdominal segment contained two D–shaped closed, dark black-colored, spiracular plates (spl) with radially arranged peritremes in the deep pit(Fig. 4D).The head of third-stage larvae comprised two antennary lobes (al) (Fig. 4E).The papillae distributed at the posterior spiracles of the third-stage larvae of *C. titillator* exhibited delineated cuticle (asterisk) and a raised circular area from which the sensory papillae originated centrally (ra). The papillae were button-shaped (pa) (Fig. 4F). The large fleshy spines (f) exhibited a tapered end, while small spines (ss) were behind the fleshy spines. This last segment is formed from dorsal (dl) and ventral lips (vl). The ventral lips contained several sensory papillae (sp) at its surface and small spines, anal orifice (an), anal papillae(ap), and crystal scar (es) (Fig. 4E,G,D and H).

### Life cycle brief description

**Fig. 5:**
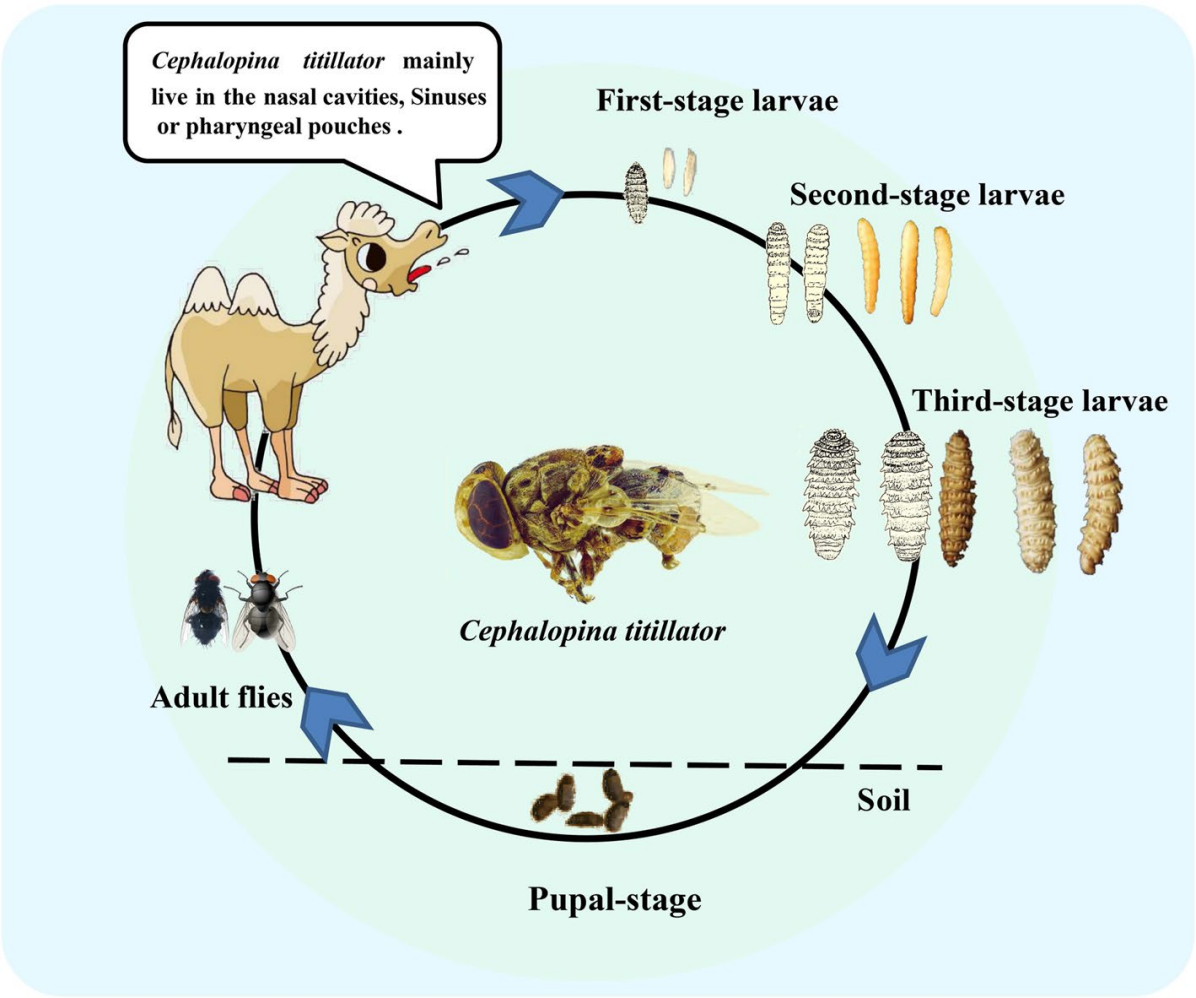
The life cycle of *C. titillator*

Adult flies appear from June to September every year. As shown in Fig. 5, the females and males land on the stems and leaves of plants to mate or mate in flight. The eggs are formed in the female flies. Subsequently, adults live freely and gather on the heads of the camels. The females lay eggs around the nostrils of camels. Eggs hatch spontaneously in less than a week. The first-stage larvae burrow through the sinuses and throat and migrate through the nasal cavity of the host. The larvae grow and become second-stage larvae after 8 or 9 months of parasitism in camels. After reaching full development, the third-stage larvae continue to crawl down into the mucous membrane of the nasal passage. The fully developed third-stage larvae are expelled via sneezing and enter the soil to pupae. The pupae emerges into flies under suitable conditions.

### Prevalence of C. titillator in camels

To date, there are no new reports on the prevalence of *C. titillator* infestation in Bactrian camels (Table 1). From March 2019 and March 2021, a total of 1263 camels from four sampling sites were detected in three geographical regions of northwestern Xinjiang. Of these, 685 camels (overall prevalence 54.2%, 95%CI: 51.44–57.01%) were infested with the *C.titillator* larvae. The infestation was detected in all four camel pastures at a prevalence rate of 38.7% (95%CI: 33.96–43.68%) at Kitagel Town Pasture, 63.6% (95%CI: 57.89–68.87%) at Karamagai Town Pasture, 67.2% (95%CI: 62.52–71.59%) at Bestierek Town Pasture and 35.8% (95%CI: 27.43–45.15%) at Qibal Town Pasture. There is statistically significant difference (χ^2^ = 41.70; *P* < 0.05) variations detected in prevalence among the four pastures of camels in the Altay region of Xinjiang. Logistic regression revealed that camels from Karamagai Town Pasture (OR = 0.37; 95%CI: 0.27–0.49; *P* < 0.001) and Bestierek Town Pasture (OR = 0.31; 95%CI: 0.23–0.41; *P* < 0.001) were more likely to be infested with *C. titillator* larvae than Kitagel Town Pasture (Table 2).

**Table 1:** Prevalence of *Cephalopina titillator* infestations in the World in studies published in the past two decades

Country	Study period	Host	No. examined	Prevalence (%)	Reference
China (Alxa Left Banner)	1989	*Camelus Bactrianus*	60	28.3	[10]
China (SouthernAlxa Left Banner)	1995	*Camelus Bactrianus*	150	18	[10]
China (Bugutusumu, Left banner)	1997	*Camelus Bactrianus*	114	18.4	[34]
China (Ulanqab, Bayannaoer, and Alxa)	2008–2009	*Camelus Bactrianus*	149	42.3	[34]
Jordan (Four geographic regions)	1996–1998	*Camelus dromedarius*	525	33.00	[11]
Ethiopia (Somali State)	1997–1998	*Camelus dromedarius*	778	71.70	[35]
Saudi Arabia (Riyadh)	1999–2000	*Camelus dromedarius*	860	41.00	[28]
Iran (eastern)	2007–2008	*Camelus dromedarius*	1328	58.10	[36]
Tehran	NG	*Camelus dromedarius*	50	100.00	[37]
Libya (Western)	2007–2008	*Camelus dromedarius*	589	79.00	[12]
Iran (Najaf-Abad)	2007–2008	*Camelus dromedarius*	384	80.72	[38]
Iraq (Al-Diwaniya city)	2008–2009	*Camelus dromedarius*	820	42.43	[6]
Egypt (Qalyubia Governorate)	2011–2012	*Camelus dromedarius*	240	41.67	[16]
Sudan (Ombadda and Tambul localities)	2012–2013	*Camelus dromedarius*	537	55.86	[39]
Ethiopia (Dire Dawa Administrative Region)	2013–2014	*Camelus dromedarius*	402	81.10	[40]
Iran (Yazd province)	NG	*Camelus dromedarius*	300	52.30	[19]
Ethiopia (Addis Ababa abattoir)	2016–2017	*Camelus dromedarius*	334	82.60	[26]
Jordan (Ramtha slaughterhouse)	1999–2000	*Camelus dromedarius*	97	46.39	[41]
Iraq (Southern)	2015–2016	*Camelus dromedarius*	864	40.07	[26]
Egypt (Cairo and Giza governorates)	2017	*Camelus dromedarius*	250	35.2	[42]

**Table 2:** Prevalence of *Cephalopina titillator* infestations among camels in the examined sampling sites

Sampling sites	Statistical analysis			95%CI	χ^2^(*P-*value)	OR(95%CI for OR)	*P* value
	No. Examined	No. Positive	Prevalence (%)				
Kitagel Town Pasture^a^	403	156	38.7	33.96–43.68	41.70(0.02)	1	
Karamagai Town Pasture	310	197	63.6	57.89–68.87		0.37(0.27–0.49)	< 0.001
Bestierek Town Pasture	430	289	67.2	62.52–71.59		0.31(0.23–0.41)	< 0.001
Qibal Town Pasture	120	43	35.8	27.43–45.15		1.13(0.74–1.73)	0.57
Total	1263	685	54.2	51.44–57.01			

*OR* Odds ratio, *CI* Confidence interval

^a^Reference category

The association between *C. titillator* infestation and risk factors (gender, season, husbandry methods, and age) of the study animals are summarized in Table 3. Out of 251 male and 1012 female camels examined 52.6% (95%CI: 46.22–58.88%) and 54.6% (95%CI: 51.51–57.73%) were infested by *C. titillator* larvae respectively. There was no significant difference (χ^2^ = 0.34; *P* > 0.05) between different sex groups of camels. Compared with that in the warm seasons (48.4%; 95%CI: 44.84–51.90%), the *C. titillator* infestation rate was significantly higher in the cold seasons (64.2%; 95%CI: 59.63–68.49%) (χ^2^ = 29.39; *P* < 0.001). Logistic regression analysis also revealed that camels in the colder seasons were more likely to be infested with *C. titillator* larvae than in the warm seasons (OR = 0.52; 95%CI: 0.41–0.66; *P* < 0.001). The infestation rate in nomadic method (47.5%; 95%CI: 44.11–50.99%) was significantly lower than that in non-nomadic method (67.2%; 95%CI: 62.52–71.59%) (χ^2^ = 44.21; *P* < 0.001). Logistic regression showed that camels non-nomadic camels were more likely to be infested with *C. titillator* larvae than in the nomadic camels (OR = 2.95; 95%CI: 1.87–4.65; *P* < 0.001). Additionally, the *C. titillator* infestation rates varied among various age groups. The prevalence of *C. titillator* infestation in camels aged < 5 years (31.7%; 95%CI: 26.34–37.51%) was significantly lower than that in camels aged 5 to 10 (60.1%; 95%CI: 55.35–64.75%) and > 10 years (61.1%; 95%CI: 56.89–65.21%) (χ^2^ = 74.23; *P* < 0.001). Logistic regression revealed that camels of aged 5 to 10 years old (OR = 0.31; 95%CI: 0.22–0.42; *P* < 0.05) and aged > 10 years old (OR = 0.30; 95%CI: 0.22–0.40; *P* < 0.001) were more likely to be infested with *C. titillator* larvae than those of aged < 5 years old.

**Table 3:** Prevalence and risk factors association with occurrence of *Cephalopina titillator*

Variables		Statistical analysis			95%CI	χ^2^(*P-*value)	OR(95%CI for OR)	*P* value
		No. Examined	No. Positive	Prevalence (%)				
Gender	Males^a^	251	132	52.6	46.22–58.88	0.34(0.56)	1	
	Females	1012	553	54.6	51.51–57.73		1.10(0.63–1.91)	0.75
Seasonal variation	Warm seasons^a^	794	384	48.4	44.84–51.90	29.39(< 0.001)	1	
	Cold seasons	469	301	64.2	59.63–68.49		0.52(0.41–0.66)	< 0.001
Animal husbandry method	Nomadic^a^	833	396	47.5	44.11–50.99	44.21(< 0.001)	1	
	Non-nomadic	430	289	67.2	62.52–71.59		2.95(1.87–4.65)	< 0.001
Camel age(year)	< 5^a^	281	89	31.7	26.34–37.51	74.23(< 0.001)	1	
	5–10	434	261	60.1	55.35–64.75		0.31(0.22–0.42)	0.02
	> 10	548	335	61.1	56.89–65.21		0.30(0.22–0.40)	< 0.001

*OR* Odds ratio, *CI* Confidence interval

^a^Reference category

### Sequencing and homologous comparison analysis

**Fig. 6:**
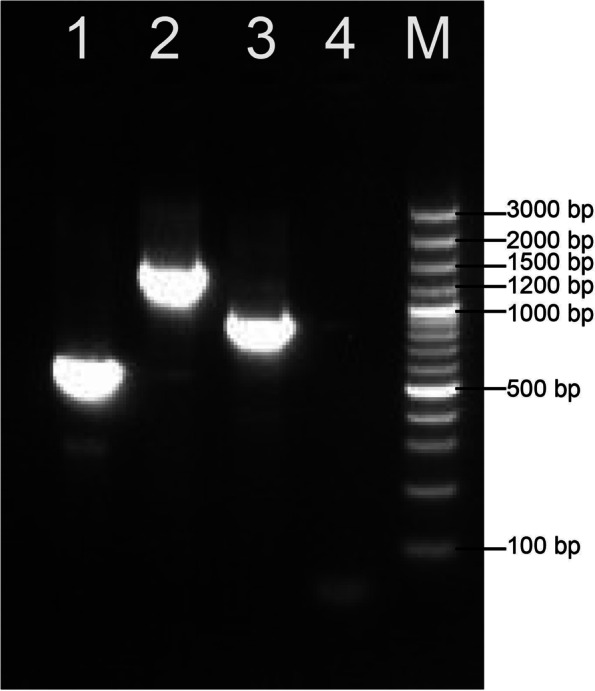
Agarose gel showing PCR amplification of *C. titillator*. Lane 1: partial *COX1* gene; Lane 2: partial *COX1* gene; Lane 3: partial *CYTB* gene; Lane 4: Negative control; Lane M: DNA marker

**Fig. 7:**
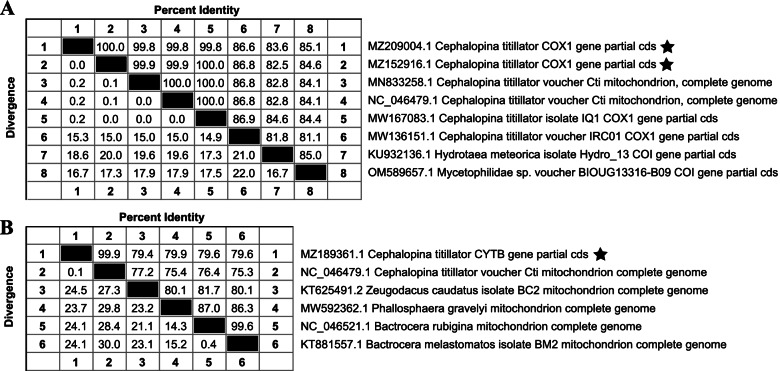
Homologous comparison of nucleotide sequences of *COX1* (**A**) and *CYTB* (**B**) genes

PCR amplification of *C. titillator COX1* and *CYTB* specific primers yielded products with electrophoresis bands of expected sizes (Fig. 6). Sequencing of *COX1-1, COX1-2* and *CYTB* yielded nucleotide sequences of approximately 552 bp, 1372 bp, and 915 bp respectively. All larval *COX1* and *CYTB* genes isolated from camels in different regions exhibited the same DNA sequence, only one of the larval sequences was selected in the subsequent analyses. Standard BLAST analysis revealed that the *COX1* sequences of *C. titillator* exhibited 99.81% and 99.92% identities with those submitted in the GenBank database (MW167083.1, NC_046479.1). The *CYTB* sequences of *C. titillator* exhibited 99.88% identities with those submitted in the GenBank database (NC_046479.1). Multiple sequence alignment of *COX1* and *CYTB* genes and homologous comparison analysis (Fig. 7) revealed that the larvae recovered from local Bactrian camels were genetically closely related to *C. titillator* recovered from the dromedary camel. All suspected samples were molecularly identified as *C. titillator*. Grossly, the results of molecular detection were consistent with those of morphological analysis. The obtained sequences were deposited in GenBank (MZ152916.1 and MZ209004.1 for *COX1*; MZ189361.1 for *CYTB*).

## Discussion

Parasitic infections have piqued the interest of the global scientific community. Camel nasopharyngeal myiasis, a common and widely distributed parasitic infectious disease that is caused by *C. titillator*, is associated with economic losses to the camel industry [42]*.* Compared with other livestock species, Bactrian camel slaughtering is difficult owing to the animal size, body structure, and labor-intensive processes [43]. A considerable amount of literature has been published on the prevalence of *C. titillator* in dromedary camels. However, there are limited studies examining *C. titillator* larvae in Bactrian camels worldwide. Over the last couple of decades, *C. titillator* has been repeatedly reported in camels from the camel-rearing areas, which indicates that this parasite is common and globally distributed (Table 1). The findings of this study firstly indicate that nasopharyngeal myiasis is widespread in areas where Bactrian camels from China. To identify the risk factors for nasopharyngeal myiasis in camels, epidemiological examinations and statistical analyses were performed. The overall infestation rate among 1263 inspected camels was 54.2%, which was higher than that reported in a previous study in Inner Mongolia [8]. This variation in the prevalence of *C. titillator* infestation may be attributed to the short study duration and the limited number of camels analyzed in earlier studies.

The previous studies revealed that the infestation rate of *C. titillator* in dromedary camels was affected by a variety of factors including age [26, 35, 36, 38, 40], gender [35, 36, 40], geographic conditions [13, 38], body condition [13, 40] and season [35, 36, 41]. The present study showed that the percentage of infested camels in the arid pastures (Karamagai Town Pasture, 63.6%; Bestierek Town Pasture, 67.2%) away from the river was higher than in the pastures (Kitagel Town Pasture, 38.7%; Qibal Town Pasture, 35.8%) near the river in the same region (*P* < 0.05). It seemed that the arid and hot conditions favour the multiplication of the flies. These results indicated that infestation rate of *C. titillator* is related to geographic conditions in which the camels reside [16]. The present study showed that no significant difference in the infestation rate of *C. titillator* (*P* > 0.05) was observed between males (52.6%) and females (54.6%). This result is inconsistent with some dromedary camels prevalence studies [13]. The reasons for these differences could be because of different management practices and environmental conditions in these camel-keeping regions [38]. The prevalence of infestation was significantly high in cold (64.2%) than in warm (48.4%) seasons (*P* < 0.001). This finding agrees with the prevalence reported in dromedary camels [36, 41]. This may be due to the intensive management and feeding of camels during cold seasons. Furthermore, the *C. titillator* infestation rate in camels fed using the nomadic method (47.5%) was lower than that in camels fed using the non-nomadic method (67.2%) (*P* < 0.001). This variation in the prevalence of *C.titillator* infestation might be the intensive feeding of camels under non-nomadic methods is more intensive, and the amount of exercise is less, and the immunity is weakened. In this study, adult camels of 5 to 10 (60.1%) and > 10 (61.1%) years of age had a significantly higher (*P* < 0.001) prevalence of *C. titillator* larvae infestation respectively as compared to young camels of < 5 (37.1%) years of age. This result is consistent with that of previous studies [12, 38, 44, 45]. Adult camels with matured body are more tolerant to external infestation and allow the deposition of egg around the nostrils [35, 38].

*C. titillate* larvae infest the posterior pharyngeal pouch and nasal mucosa of camels, causing a series of chronic lesions [46]*.* The larval conical spines may cause mucosal damage of the nasopharynx while they are attaching and detaching in different areas of the mucous membrane [35]. The nodules that contain pus and the ulcers are due to invasion by secondary bacteria. The histopathological alterations are in accordance with the previous reports that desquamation of the epithelial cells with infiltration of different types of leukocytes in the inflamed areas [46].

Electron microscope provided a new perspective on morphological characterization of *C. titillator* larval stages. This is consistent with previous research that reported that the spiracular plate of third-stage *C. titillator* larvae comprised several respiratory holes [36, 47]. These respiratory holes are prominent and scattered irregularly within the spiracular plate. The number of larval respiratory holes of different hosts has a significant taxonomic value [36]. Additionally, our observations of larval morphology are consistent with those of Marwa et al. [48] who found that the whole body of *C. titillator* larvae has sensory papillae and sensilla, which play a vital role in their behavior for deposited larvae.

The life cycle *C. titillator* generally resemble those of O. ovis [49]. The botflies are highly host-specific and site-specific parasites in the larval stage and begin to reproduce in the adult stage. Here we experimentally collected samples of three stages of larvae, pupae, and adult fly. All three larval instars were detected in infested camels each month of the year. When fully developed, the larvae enlarge their breathing holes, emerge through them, and fall to the ground to pupate. Adult flies emerge from pupal cases and mainly inhabit the vegetation in the primary stage of emergence [50]. Subsequently, matured male files can mate with female flies.

Recent studies have analyzed the genome structure, base composition, substitutional, evolutionary rates, and comprehensive molecular phylogenetic characteristics of mitochondrial genomes from the subfamily Oestrinae. The analysis revealed that the mitochondrial genome is a potential tool for application in phylogenetic analysis of Oestridae [31, 33]. In this study, mitochondrial *COX1* and *CYTB* sequences of *C. titillator* larvae were amplified and sequenced. *COX1* sequences in *C. titillator* obtained from infested Bactrian camels exhibited 99.81% (NC_046479.1, China and MW167083.1, Iraq) and 99,92% (NC_046479.1, China) identities with those obtained from dromedary camels. *CYTB* sequences of *C. titillator* exhibited 99.88% (NC_046479.1, China) identities with those obtained from dromedary camels. Morphological and molecular analyses revealed that the pathogen isolated from the Bactrian camels was *C. titillator* larvae. Therefore, we propose a hypothesis that *C. titillator* may parasitized the their common ancestor before species divergence [43]. Furthermore, the genes sequence of these parasites exhibited limited variations, which may suggest that ancient trading routes lead to the transmission of parasitic diseases among distant camel populations.

Host-parasite relationships are complex, and for both organisms a great number of specialized resistance mechanisms are involved [29]. The infested camels also develop several but often ineffective strategies of expelling parasites through lay the foundation coughing and sneezing, mucus hypersecretion, and nasal discharge [51]. The study on the *C. titillator* larvae of Bactrian camels in the Asian region, including the pathogen diagnostic, preventive, and control strategies will be performed in the future based on studies on *C. titillator*-infested dromedary camels*.*

In conclusion, this study investigated the prevalence of *C. titillator* infestation in Bactrian camels in China and determined the risk factors (sampling sites, gender, seasonal variation, animal husbandry method, and camel age) for *C. titillator* infestation. Additionally, larval morphology, gross and microscopic lesions, and life cycle of *C. titillator* were elucidated*.* The genetic distance between *C. titillator* infesting Bactrian camels and that infesting dromedary was determined using *COX1* and *CYTB* sequences. *C. titillator* infesting domestic Bactrian camels was most closely related to *C. titillator* infesting the western dromedary camels. The DNA sequence of this pathogen exhibited minimal variation. This study also shows that many positive camels have pathological lesions that difficult to observe. These lesions are caused by persistent irritation and the feeding behavior of a certain number of larvae.

This is the first study to report *C. titillator* infestation among Bactrian camels in Xinjiang, which will provide a baseline for further epidemiological and parasite control studies in the region. Additionally, the results presented in this study will lay the foundation for the diagnosis, prevention, and control strategies of *C.titillator* infestation in camels. Future studies must determine the infestation mechanism, physiology, and diagnostic markers of *C. titillator* infested camels, as well as develop preventive and control procedures for nasopharyngeal myiasis.

## Conclusion

The *C. titillator* was prevalent in Bactrian camels from all studied pastures in Xinjiang. The prevalence of *C. titillator* in camels was significantly associated with age, season, pasture environment and animal husbandry methods. This study provides valuable information for establishing surveillance programs and basic data for future research on *C. titillator* prevention and control measures of camels.

## Methods

### Study areas

**Fig. 8:**
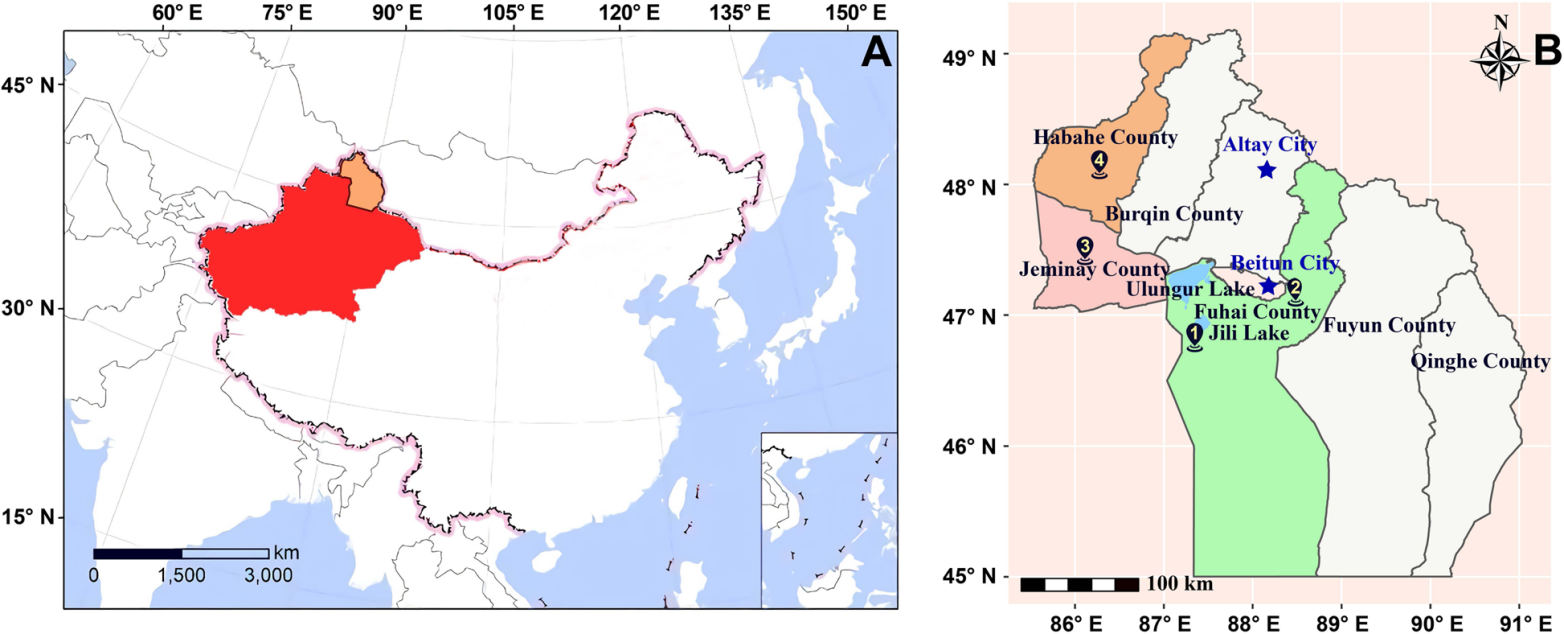
*C*.*titillator* sampling sites. **A** Location of Aletai Prefecture in Northern Xinjiang; **B** The main collection areas of *C. titillator*

This study was conducted at the Aletai prefecture of Xinjiang province situated in Northwestern China (Fig. 8A). Aletai prefecture, which has the highest number of camels in Xinjiang, has a typical temperate continental cold climate. This area is characterized by wide temperature fluctuations in all seasons and within a single day. As the Aletai prefecture is in an arid and semi-arid belt in the typical temperate continental climate zone, this area has cold and dry winters and warm and dry summers. Compared with other domestic animals, camels play a vital role in the lifestyle of many communities owing to their adaptation to extremely harsh climate conditions. The *C. titillator* of multiple stages were collected from the following four pastures: Kitagel Town (No. 1 area in Fig. 8B) (87° 35’25”E, 46° 86’6”N), Karamagai Town (No. 2 area in Fig. 8B) (88° 24’11”E, 47° 9’8”N) in Fuhai County, Bestierek Town (No. 3 area in Fig. 8B) (86° 12’9’E, 47°40’34”N) in Jeminay County, and Qibal Town (No. 4 area in Fig. 8B) (86° 20’56”E, 48° 3’4”N) in Habahe County. Camels were slaughtered in the official slaughterhouse of Fuhai and Habahe Counties.

### Sample collection

This study randomly examined 1263 Bactrian camels (*Camelus bactrianus*; 1012 males and 251 females) between March 2019 and March 2021. Both native camels and camels brought from different areas of China were included in the study cohort. The camels of Fuhai and Habahe Counties were maintained under a primitive culture model and were owned by local herders. Meanwhile, camels in the Bestierek Township of Jeminay County were maintained in captivity. The camels were an essential source of milk and meat. All infested camels were classified into the following three age groups based on tooth wear pattern: < 5, 5–10, and > 10 years [19]. The presence of *C. titillator* larvae was examined each month during the study period. The seasons of the study area were classified into the following groups: cold (September to February) and warm seasons (March to August) [12]. The antiparasitic treatment for camel nasal botfly has not been examined previously. Routine camel feeding practices and maintenance conditions were followed in this area. None of the camels were injected with any drugs to treat *C. titillator* infestation. Currently, *C. titillator* infestation is diagnosed based on gross, etiological, and histopathological examinations of collected specimens. The larvae squirted from the nose or mouth were collected from the head of the camels. Additionally, the head of slaughtered camels was subjected to sagittal incision to expose regions of nasal and pharyngeal cavities, labyrinth of the ethmoid bone, turbinates, inferior meatus, and pharynx after its separation from the rest of the body. Larvae were picked using a teasing needle and recorded the number of infested camels. Next, the larvae were washed twice with salt water and stored in 75% alcohol and 2.5% glutaraldehyde solution at 4 °C for 48 h [48]. The body sizes (length and width) of all stages of larvae, pupa, and adults were measured with a ruler.

### Statistical analysis

The geographical environmental conditions of each pasture and the factors related to the possible epidemic of the disease and their roles in the epidemic of *C. titillator* were examined.

The prevalence of infestation in different pastures and its correlation with the independent variables (gender, season, husbandry methods, and different age groups of camels) were initially analyzed using the Chi-square test [52, 53]. Binary logistic regression analysis was then performed on parameters considered significant in statistical analysis to investigate the associations between *C. titillator* larvae infestation status and pasture location, gender, season, animal feeding method, and age of the study camels [40, 54]. All statistical analyses were performed using SPSS program version 26 (IBM, USA). Differences were considered significant at *P* < 0.05.

### Histopathological examination

Representative specimens of the nasal and pharyngeal cavities, frontal sinuses, and turbinate bone were obtained from infested and non-infested camels. The tissues were fixed in 10% neutral buffered formalin, dehydrated in a graded alcohol series, cleared in xylol, embedded in paraffin, and sectioned to a thickness of 5 μm. The sections were stained with hematoxylin and eosin (H&E) following the protocols of a previous study [55] and imaged using a light microscope (Eclipse E100 Nikon, Japan).

### Morphological identification

The parasites and their body parts, such as head, mouthparts, thorax, and abdomen were observed under a stereo microscope (SMZ25, Nikon, Japan) to examine the morphology and developmental stages of the parasite [26, 33, 47, 50, 56]. The identification of the collected samples was carried out using the morphological keys described by Zumpt, et al. [1].

### Scanning electron microscopy

Fixed larvae at different stages were washed thrice with 0.1 M phosphate buffer (PB, pH 7.4) (15 min/wash). The samples were incubated with 1% OsO_4_ in 0.1 M PB (pH 7.4) for 1.5–2 h at 18–24 °C. After washing the samples with 0.1 M PB (pH 7.4) three times (15 min/step), the samples were dehydrated in a graded ethanol series and incubated with isoamyl acetate for 15 min. The samples were dried using a critical point drier. The dried samples were attached to metallic stubs using carbon stickers and sputter-coated with gold for 30 s. The micrographs were captured using a scanning electron microscope (SU8100, HITACHI, Japan) [47, 48].

### Molecular identification

*C. titillator* larvae were subjected to molecular analyses, following the protocols of Attia and Hafedh [32, 33, 56]. Total DNA of suspected larvae was extracted using a commercial Ezupcolumn animal genomic DNA purification kit (Sangon Biotech, China), following the manufacturer’s instructions and stored at − 20 °C until analysis. The following primers were designed using Primer 6.0: *COX1-1*, 5’-ATTAATTCGAATAGAGCTAGGACAC-3’ (forward)and 5’-AATGATGTGTTTAAATTCCGGTC-3’ (reverse); *COX1-2*, 5’-ATTTATAATGTAATTGTCACCGCAC-3’ (forward) and 5’-TTGGTAATTCGGCATATCTGTGT-3’ (reverse); *CYTB*, 5’-GATTATTCCTCGCCATACATTACAC-3’ (forward) and 5’-TTTGTCCTGTTATGATAAAGGGGTT-3’ (reverse). PCR was performed in a 25 μL reaction volume comprising 2.5 μL of 10 × Taq buffer supplemented with MgCl_2_, 0.2 μL of Taq DNA polymerase (5 U/μL), 1 μL of extracted DNA template, 1 μL of each primer (10 μM), 1 μL of dNTP mix (10 μM), and sterile deionized water. The PCR conditions were as follows: initial holding temperature of 95 °C for 5 min, followed by 10 cycles at 94 °C for 30 s, 63 °C (decrease 0.5 °C per cycle) for 30 s, 72 °C for 30 s; followed by 30 cycles at 95 °C for 30 s (denaturation), 58 °C for 30 s (annealing), and 72 °C for 30 s (extension) and a final extension step at 72 °C for 10 min. The amplicons were analyzed using agarose gel electrophoresis on a 1% gel, purified using a SanPrepcolumn DNA gel extraction kit (Sangon Biotech, China), and outsourced to Shanghai Sangon Biological Engineering Technology and Services Company for paired-end sequencing. Nucleotide sequences of the *COX1* and *CYTB* was aligned to the closely related sequences listed in GenBank using the Basic Local Alignment Search Tool (BLAST). Meanwhile, the homologous comparison of nucleotide sequences analysis was performed using the Clustal W method with Megalign software. 

## Supplementary Information

**Additional file 1.** 

## Data Availability

The datasets generated and/or analyzed during the current study are available from the corresponding author on reasonable request.

## Abbreviations

C. titillator: Cephalopin atitillator
CI: Confidence Interval
PCR: Polymerase Chain Reaction
SEM: Scanning Electron Microscope
*COX1*: Cytochrome c oxidase subunit 1
*CYTB*: Cytochrome B gene

## References

[1] Zumpt F (1965). Myasis in Man and Animals in the Old World. A Textbook for Physicians, Veterinarians and Zoologists. A Textbook for Physicians, Veterinarians and Zoologists.

[2] Hussein MF, Elamin FM, El-Taib NT. Basmaeil SMJVP: The pathology of nasopharyngeal myiasis in Saudi Arabian camels (Camelus dromedarius). Vet Parasitol. 1982;9(3–4):179–83. 10.1016/0304-4017(82)90060-7 7201200

[3] Higgins AJ (1985). Common ectoparasites of the camel and their control. Brit Vet J.

[4] Hall MJR, Wall R (1995). Myiasis of humans and domestic animals. J Adv Parasitol.

[5] Otranto D. The immunology of myiasis: parasite survival and host defense strategies. Trends Parasitol. 2001;17(4):176–82. 10.1016/s1471-4922(00)01943-7 11282507

[6] Atiyah W (2011). Cephalopina titillator.

[7] Desbordes OK, Ajogi I. Seasonal prevalence of Cephalopina titillator myiasis in camels (Camelus dromedarius) in Sokoto State. Nigeria Vet Parasitol. 1993;50(1–2):161–4. 10.1016/0304-4017(93)90018-i 8291193

[8] Li YZ, Yang XY, Zhu YW, Xing ZG, Wu HY, Tang SM, Hao LJ, Baiyinwuliji, City H: Epidemiological investigation of immature Cephalopina titillator infestation in Bactrian camels. Chinese Veterinary Science 1997;27(12):11-2.

[9] Ramadan MY (1997). Studies on some ectoparsites of camels.

[10] Li YZ, Zhu YW, Wu HY, Xing ZG, Yang XY, Tang SM. WLJ B: Investigation of Cephalopod titillate maggot infection rate and intensity in Bacteria camel. Inner Mongolia Animal Husbandry Science. 1998;(01):43-5.

[11] Al-Rawashdeh O, Al-Ani FK, Sharrif LA, Al-Qudah KM, Frank N. A survey of camel (Camelus dermedarius) diseases in Jordan. J Zoo Wildlife Med. 2000;31(3):335–338. 10.1638/1042-7260(2000)031[0335:ASOCCD]2.0.CO;2 11237140

[12] Sanchez SE. Prevalence and pathology of nasal myiasis in camels slaughtered in El-Zawia Province-Western Libya: with a reference to thyroid alteration and renal lipidosis. Global Veterinaria. 2010;4(2):190–197.

[13] Kissi LM, Assen AM (2017). Prevalence, Larvae Burden and Gross Pathological Lesion of Cephalopina titillator in Camels Slaughtered at Addis Ababa Abattoir Akaki Branch, Ethiopia. J Vet Sci Tech.

[14] Punsmann TM, Grimm LM, Reckmann C, Schwennen C (2018). Research SJBV: First report on nasal myiasis in an alpaca "Vicugna pacos" - a case report. Bmc Vet Res.

[15] Yousef HA, Afify A, Meguid AA, Hassan HM (2015). Profiling of proteins and proteases in the products of the salivary gland, digestive tract and excretions from larvae of the camel nasal botfly, Cephalopina titillator (Clark). Z Naturforsch C.

[16] Khater HF, Ramadan MY, Mageid A. In vitro control of the camel nasal botfly, Cephalopina titillator, with doramectin, lavender, camphor, and onion oils. Parasitol Res. 2013;112(7):2503–10. 10.1007/s00436-013-3415-2 23604566

[17] Khater HF (2014). Bioactivities of some essential oils against the camel nasal botfly Cephalopina titillator. J Parasitology Res.

[18] Chhabra MB, Sangwan AK (2006). Parasitic diseases of camels - An update 1 Protozoal diseases. J Camel Pract Res.

[19] Jalali MHR, Dehghan S, Haji A, Ebrahimi M. Myiasis caused by Cephalopina titillator (Diptera: Oestridae) in camels (Camelus dromedarius) of semi-arid areas in Iran: distribution and associated risk factors. Comp Clin Pathol. 2016;25(4):677–80.

[20] Sazmand A, Joachim A (2017). Parasitic diseases of camels in Iran (1931–2017)-a literature review. Parasite.

[21] Fetene E, Leta S, Regassa F, Büscher P: Global distribution, host range and prevalence of Trypanosoma vivax: a systematic review and meta-analysis. Parasite Vector. 2021;14(1):1-20. 10.1186/s13071-021-04584-x PMC7830052 33494807

[22] Zhao SSLY, Zhang Y, Zhou Q, Jing B, Xu CY, Zhang LX, Song JK, Qi M, Zhao GH. Multilocus genotyping of Giardia duodenalis in Bactrian camels (Camelus bactrianus) in China. Parasitol Res. 2020;119(11):3873–80. 10.1007/s00436-020-06905-y 33006040

[23] X Chen, Q Yao, DJ Wang, KQ Meng, XU CM, HE XM, WU LH, LI GZ, Song J: Investigation on Gastrointestinal Nematode Infections of Bactrian Camels in Partial Areas of Alxa Left Banne,Inner Mongolia. Progress in Veterinary Medicine. 2019;40(10):130-3.

[24] Li ZZX, Tian WL, Chen JZ, Guo YT, Hu XY, Li SN, Tian RX, Dong WL, Su ZQ, Yao G, Ran DL, Fu Q, Shi HJ (2020). Investigation of parasitic infection in the digestive tract of Bactrian camels in some areas of Xinjiang. Heilongjiang Anim Sci Veterinary Med.

[25] Zhou H, Ma Z, Hu T, Bi Y, Shi W: Tamdy Virus in Ixodid Ticks Infesting Bactrian Camels, Xinjiang, China, 2018. Emerg Infect Dis. 2019;25(11):2136-8. 10.3201/eid2511.190512 PMC6810205 31625865

[26] Al-Jindeel T, Jasim HJ, Alsalih NJ, Al-Yasari A: Clinical, Immunological and Epidemiological Studies of Nasopharyngeal Myiasis in Camels slaughtered in Al-Muthanna Province. Adv Anim Vet Sci. 2018;6(7):299-305.

[27] AL-Nasr I, El-Bahy M, Al-Dubib M. Characterization of a Specific Purified Protein Fraction for Diagnosis of Cephalopina Nasal Myiasis in Camels in Saudi Arabia. Asian J Sci Technol. 2013;4(5):032–7.

[28] Al-Ahmed A (2002). Seasonal Prevalence of Cephalopina titillator Larvae in Camels in Riyadh Region, Saudi Arabia. Arab Gulf J Sci Res..

[29] Yousef HAMA, Afify A, Hassan HM (2016). Analysis of larval antigens of Cephalopina titillator in the camel mucus for diagnosis of infestation. Biologia.

[30] Toaleb NI, Abdel-Rahman EH. Accurate Diagnosis of Camel Nasal Myiasis using Specific Second Instar Larval Fraction of Cephalopina titillator Extract. Adv Anim Vet Sci. 2020;9(5):674–81.

[31] Li XY, Yan LP, Pape T, Gao YY, Zhang D. Evolutionary insights into bot flies (Insecta: Diptera: Oestridae) from comparative analysis of the mitochondrial genomes. Int J Biol Macromol. 2020;149:371–80. 10.1016/j.ijbiomac.2020.01.249 31991213

[32] Hendawy S, Allam N, Kandil OM, Zayed AA, El-Seify MA. Partial COI and 16S rRNA Genes Sequences of Cephalopina titillator Mitochondrial DNA: Evidence for Variation in Evolutionary Rates within Myiasis-Causing Species. Global Veterinaria. 2012;9(6):69–778.

[33] Hafedh AA, Humide AO, Alrikaby NA. Cytochrome Oxidase Subunit I (COI) Gene Sequencing for Identification of Cephalopina Titillator local Isolates from Camels in Thi-Qar Province/Iraq. Int J Trop Insect Sc. 2021;25(4):3146–52.

[34] Hendawy S, Allam N, Kandil OM, Zayed AA, El-Seify MA: Immunological Evaluation of the Diagnostic Values of Cephalopina titillator Different Larval Antigens in Camels from Egypt. Global Veterinaria. 2013; 10(2):158-64.

[35] Bekele T, Molla B (2001). Mastitis in lactating camels (Camelus dromedarius) in Afar Region, north-eastern Ethiopia. Berl Munch Tierarztl Wochenschr.

[36] Oryan A, Valinezhad A, Moraveji M. Prevalence and pathology of camel nasal myiasis in eastern areas of Iran. Trop Biomed. 2008;25(1):30–6. 18600202

[37] Qiu C, Sanchez SE, Gelaye B, Enquobahrie DA, Ananth CV, Williams MA: Prevalence and pathology of nasal myiasis in camels slaughtered in El-Zawia Province-Western Libya: with a reference to thyroid alteration and renal lipidosis. Global Veterinaria. 2010;28:1-6.

[38] Shakerian A, Hossein SR, Abbasi M. Prevalence of Cephalopina titillator (Diptera: Oestridae) larvae in one-humped camel (Camelus dromedarius) in Najaf-Abad. Iran Global veterinaria. 2011;6(3):320–3.

[39] Makin E (2015). Detection and Prevalence of Nasal Bot Fly (Cephalopina titillator) Larvae in Camels in Slaughterhouses in Ombadda and Tamboul Localities, Sudan.

[40] Mumed A, Gemeda AE. A Cross Sectional Study on Prevalence of Cephalopina titillator Infection in Camel (Camelus dromedaries) in Dire Dawa Administrative Region. Ethiopia Advances in Biological Research. 2015;9(4):225–9.

[41] Al-Ani F. Seasonal prevalence of the larvae of the nasal fly (Cephalopina titillator) in camels in Jordan. Revue d’élevage et de médecine vétérinaire des pays tropicaux. 2016;69(3):125–7.

[42] Abu E, Hassan N, Namaky AE, Abo-Aziza F (2018). Efficacy of some essential oils on Cephalopina titillator with special concern to nasal myiasis prevalence among camels and its consequent histopathological changes. J Parasit Dis.

[43] Burger PA, Ciani E, Faye B (2019). Old World camels in a modern world – a balancing act between conservation and genetic improvement. Anim Genet.

[44] Fatani A, Hilali M. Prevalence and monthly variations of the second and third instars of Cephalopina titillator (Diptera: Oestridae) infesting camels (Camelus dromedarius) in the Eastern Province of Saudi Arabia. Vet Parasitol. 1994;53(1–2):145–51. 10.1016/0304-4017(94)90026-4 8091611

[45] Bassiony G, Sagair O, Daly E, Nady A: Alterations in the Pituitary- Thyroid Axis in Camel Camelus dromedarius Infected by Larvae of Nasal Bot Fly Cephalopina titillator. J anim vet adv. 2005;4(3):345-8.

[46] Bekele T (2001). Studies on Cephalopina titillator, the Cause of 'Sengale' in Camels (Camelus dromedarius) in Semi-arid Areas of Somali State. Ethiopia. Trop Anim Health Pro.

[47] Zayed AA, Abdel-Shafy S, El-Khateeb RM (2008). Surface ultrastructure of posterior abdominal spiracles of third instars of nasal bots of Cephalopina titillator, Oestrus ovis and Rhinoestrus purpureus(Diptera: Oestridae) infesting camels, sheep and donkeys in Egypt. Res J Parasitol.

[48] Attia MM, Mahdy OA. Fine structure of different stages of camel nasal bot;Cephalopina titillator(Diptera: Oestridae). Int J Trop Insect Sc. 2021;42(1):677–84.

[49] Bowman DD, Georgi JR (2013). Georgis' parasitology for veterinarians: Georgisﻗ°ﻷ parasitology for veterinarians.

[50] El-Hawagry M, Abdel-Dayem MS, Dhafer H. The family Oestridae in Egypt and Saudi Arabia (Diptera, Oestroidea). Zookeys. 2020;947(3):113–42. 10.3897/zookeys.947.52317 PMC7363712 32733132

[51] Colwell DD, Hall M, Scholl PJ (2006). The oestrid flies: biology, host-parasite relationships, impact and management.

[52] Abdulkareem BO, Christy AL, Samuel UU (2018). Prevalence of ectoparasite infestations in owned dogs in Kwara State. Nigeria Parasite Epidemiology & Control.

[53] Porsani M, Teixeira FA, Oliveira VV, Pedrinelli V (2020). Brunetto MAJSR: Prevalence of canine obesity in the city of So Paulo, Brazil. Sci Rep.

[54] Luu L, Bettridge J, Christley RM, Melese K, Lynch SE (2013). Prevalence and molecular characterisation of Eimeria species in Ethiopian village chickens. Bmc Vet Res.

[55] Layton C, Bancroft JD, Suvarna SK (2019). ixation of tissues. Bancrofts Theory & Practice of Histological Techniques.

[56] Attia MM, Farag HS, Abdel-Saeed H, Ismael E. Advanced immunological studies on Cephalopina titillator with special references to the epidemiological uses of Dot-ELISA in camel sera. J Parasit Dis. 2020;44(4):813–21. 10.1007/s12639-020-01256-y PMC7596128 33184548

