# Supplementary material for: Prevalence and pathology of Cephalopina titillator infestation in Camelus bactrianus from Xinjiang, China
Source: BMC Vet Res. 2022 Sep 28;18:360. doi: 10.1186/s12917-022-03464-5 (PMC9520952; doi:10.1186/s12917-022-03464-5)
Supplement: Supplementary file 1 — Additional file 1. [file 12917_2022_3464_MOESM1_ESM.pdf]

Supplementary material

PCR reaction system

Content	Concentration	Volume（μl）
DNA		1
F	10 μM	1
R	10 μM	1
Dntp (mix)	10 mM	1
Taq Buffer (with MgCl2)	10X	2.5
Taq	5 U/μl	0.2
add ddH2O to		25

PCR reaction conditions

Step	Program	Temperature	Time
1	Predenaturation	95°C	5 min
2	Denaturation	94°C	30 sec
3	Primer Annealing	63°C (0.5°C per cycle decrease)	30 sec
4	Primer Extension	72°C	30 sec
5	Repeat steps 2 to 4	10cycles	
6	Denaturation	95°C	30sec
7	Primer Annealing	58℃	30sec
8	Primer Extension	72℃	30sec
9	Repeat steps 6 to 8	30cycles	
10	Repair extension	72℃	10min
11	Hold	4℃	

Primer sequences

COX1-F 5' ATTAATTCGAATAGAGCTAGGACAC 3'

COX1-R 5' AATGATGTGTTTAAATTCCGGTC 3'

9-560=552

COX2-F 5' ATTTATAATGTAATTGTCACCGCAC 3'

COX2-R 5' TTGGTAATTCGGCATATCTGTGT 3'

151-1522=1372

CytB-F 5' GATTATTCCTCGCCATACATTACAC 3'

CytB-R 5' TTTGTCCTGTTATGATAAAGGGGTT 3'

146-1060=915

Electropherogram

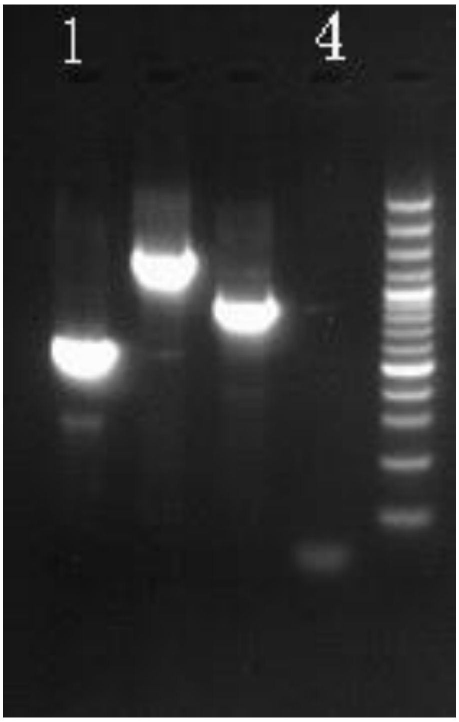

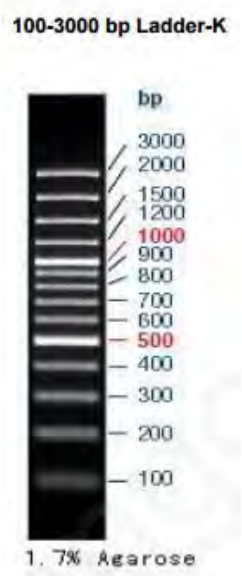

COXI

AGAGCTAGGACACCCCGGAACGCTCATTGGAAATGATCAAATTTATAATG
TAATTGTCACCACACATGCTTTCATCATAATTTTCTTTATAGTTATACCAAT
TATAATTGGAGGATTTGGAAATTGACTAGTCCCACTAATACTAGGGGCCC
CAGATATAGCATTCCCTCGAATAAATAATATAAGATTCTGACTTTTACCTC
CCGCTCTTACACTCCTTCTAACAAGAAGAATAGTAGAAAGCGGCGCTGGC
ACTGGATGAACTGTTTATCCTCCTCTTTCATCAAATATTGCCCACAGAGGA
GCCTCCGTAGATTTAGCAATTTTCTCACTTCATTTAGCTGGAATTTCATCCA
TTTTAGGAGCAGTTAATTTTATTACAACAATCATCAATATACGATCTATT
GGAATAACTTTAGATCGAACACCCTTATTTGTATGATCTGTAATAATTACA
GCAATTCTTTTACTTCTATCTCTACCAGTTTTAGCAGGAGCCATTACAATA
CTATTAACCGACCGGAATTTA

COX2
CTTTATAGTTATACCAATTATAATTGGAGGATTTGGAAATTGACTAGTCCC
ACTAATACTAGGGGCCCCAGATATAGCATTCCCTCGAATAAATAATATAA
GATTCTGACTTTTACCTCCCGCTCTTACACTCCTTCTAACAAGAAGAATAG
TAGAAAGCGGCGCTGGCACTGGATGAACTGTTTATCCTCCTCTTTCATCAA
ATATTGCCCACAGAGGAGCCTCCGTAGATTTAGCAATTTTCTCACTTCATT
TAGCTGGAATTTCATCCATTTTAGGAGCAGTTAATTTTATTACAACAATCA
TCAATATACGATCTATTGGAATAACTTTAGATCGAACACCCTTATTTGTAT
GATCTGTAATAATTACAGCAATTCTTTTACTTCTATCTCTACCAGTTTTAGC
AGGAGCCATTACAATACTATTAACCGACCGGAATTTAAACACATCATTTTT
TGACCCTGCAGGAGGGGGAGACCCCATTCTTTATCAACACTTATTTTGATT
TTTTGGACACCCCGAAGTTTACATTTTAATTCTCCCCGGATTCGGAATAAT
TTCCCATATTATTAGACAAGAATCAGGAAAAAAGGAAACCTTTGGAGCTC
TAGGAATAATCTACGCCATATTAGCCATTGGACTGTTAGGATTCATTGTTT
GAGCCCACCACATATTTACAGTTGGAATAGATATTGATACCCGAGCTTAC
TTTACTTCTGCTACAATAATCATTGCCGTTCCTACGGGAATTAAAATTTTT
AGCTGACTAGCCACACTATATGGCACCCAACTAAACTGATCTCCTGCCAT
ATTATGATCACTAGGATTCGTATTTCTATTTACAGTGGGCGGATTAACAGG
AGTAGTACTAGCTAATTCATCCGTAGACATTATATTACATGATACATATTA
TGTTGTAGCCCACTTCCATTACGTTTTATCAATAGGAGCAGTTTTCGCTAT
CATAGGAGGATTTATTCACTGATTCCCCCTATTTACTGGATTAACATTTAA
TAAAATACTTCTTCAAACACAATTTATTGTAATATTTATAGGAGTAAATTT
AACATTCTTCCCCCAACACTTCCTTGGACTCTCCGGCATACCCCGCCGATA
TTCAGATTACCCAGATGCATATACTGCTTGAAATGTAATCTCTTCAATCGG
ATCAACAATTTCATTTCTAGGAATTTTAATATTTATATATATTATCTGAGA
AAGATTAACATCTCAACGAAAAATTTTATTTTCAAATCAATTAAACTCATC
AAT

CytB
ACCATATTTGCCGGGATGTTAATTATGGATGAATTCTTCGAACCCTTCATG
CCAATGGAGCATCATTTTTTTCATCTGCATTTACTTTCACATTGGTCGAGG
AATTTATTATAACTCATATACCTTTACCCCCACATGAATAATTGGAGTAAT
TATTCTATTCCTATTAATAGCAACAGCATTTATAGGATATGTACTCCCATG
AGGACAAATATCATTTTGAGGCGCCACAGTAATTACTAATCTTCTATCAGC
AATCCCATACTTAGGGACTACTTTAGTACAATGAATTTGAGGAGGCTTCG
CAGTAGACAATGCCACTCTTACACGATTCTTTACATTCCACTTCATTCTAC
CCTTTATTGTTCTTGCCACAACTTTAATTCATATCTTATTTCTACACGAAAC
AGGATCTAATAACCCCCTAGGAATCAACTCAAACGTAAATAAAATCCCCT
TCCACCCCTACTTTACCTACAAAGACATCGTAGGATTTTCAATAATATTAA
TAGCATTGATTCTATTAACACTAATAAATCCCTACCTACTAGGAGACCCAG
ACAACTTCATCCCAGCAAACCCTCTAGTAACCCCCATTCATATCCAACCTG
AATGATACTTTTTATTCGCTTACGCAATTTTACGCTCAATCCCCAATAAAT
TAGGAGGAGTAATTGCACTAATTATATCAATTGCAATTCTAATGATTCTCC
CATTTTACCATATAAGAAAATTTCGAGGAATTCAATTCTATCCTATTAATG
AAATTCTATTTTGAACAATAATTATTACAATTATCCTACTTACA

Scanning Electron Microscopy Report

1. Apparatus and Reagents

1.1 Major Apparatus

Name	Producer	Model
Critical Point Dryer	Quorum	K850
Lon Sputtering Apparatus	HITACHI	MC1000
Scanning Electron Microscope	HITACHI	SU8100

1.2 Major Reagents

Name	Producer	Code
Fixative for TEM	Servicebio	G1102
Ethanol	Sinaopharm Group Chemical Reagent Co. LTD	100092183
Isoamyl acetate	Sinaopharm Group Chemical Reagent Co. LTD	10003128
PBS	Servicebio	G0002
OsO_4	Ted Pella Inc	

2. Procedure

2.1 **Harvest tissue block and fixation**: Targeted fresh tissues should be selected to minimize mechanical damage such as pulling, contusion and extrusion. Use a sharp blade to cut and harvest fresh tissue blocks quickly within 1-3 minutes. The area of tissue block should be no more than 3 mm^2. Wash tissues with PBS gently to remove the blood and hair, etc. Label the target side of tissue (the side you want to observe) by any way, such as making cuts on the opposite side. Make sure to protect tissue blocks, especially the target side, from mechanical damage such as forceps extrusion. The washed tissue blocks are immediately fixed by electron microscopy fixative for 2 hours at room temperature, then transferred into 4°C for preservation and transportation.

For adherent cell: Seed cells on a sterile cover glass in a petri dish. Remove the culture medium, then wash slide gently with PBS, followed by adding electron microscopy fixative into petri dish. After fixing for 2 hours at room temperature, transfer the petri dish to 4°C for preservation and transport. Note to avoid sever shock which may result in the cells dropping off from cover glass.

2.2 **Post-fix:** Wash tissue blocks with 0.1 M PB (pH 7.4) for 3 times, 15 min each. Then transfer tissue blocks into 1% OsO_4 in 0.1 M PB (pH 7.4) for 1-2 h at room temperature. After that, wash tissue blocks in 0.1M PB (pH 7.4) for 3 times, 15 min each.

2.3 Dehydrate as followed:

30% ethanol for 15 min;

50% ethanol for 15 min;

70% ethanol for 15 min;

80% ethanol for 15 min;

90% ethanol for 15 min;

95% ethanol for 15 min;

Two changes of 100% ethanol for 15 min;

Finally, isoamyl acetate for 15 min.

2.4 Drying: Dry samples with Critical Point Dryer.

Note: Dry samples and inorganic materials should ignore all the steps above and do the following step directly.

2.5 Conductive metal coating: Specimens are attached to metallic stubs using carbon stickers and sputter-coated with gold for 30s.

2.6 Observe and take images with scanning electron microscope.

Independent variables

Factors	Variable name	Assignment Description
Camels	ID	
Gender	Gender	Males =0; Females =1
Sampling sites	Sampling sites	Kitagel Town Pasture=1;<br>Karamagai Tow Pasture=2;<br>Bestierek Township Pasture=3;<br>Qibal Township Pasture=4
Camel age(year)	Camel age(year)	＜5 =1<br>5-10 =2<br>＞10 =3
Seasonal variation	Warm/cold seasons	Warm =0, cold =1
Animal husbandry methods	Nomadic/ Non-nomadic	Nomadic =0, Non-nomadic =1
Infestation	Infested/ Uninfested	Infested=1, Uninfested=0

Larval features

Stages of Larval development	Morphological Features	Parasitic Tissue Site	Time of Appearance
First stage	Body length 2.14±0.02 mm, width 0.67±0.01 mm，White and yellowish, segmented, fusiform, with clusters of small spines	Frontal sinus, paranasal sinus and turbinate, adhere to the mucosal surface with mouth hooks	Every year from July to December and from January to March of the following year
Second stage	Body length 11.62±0.09 mm, width 3.64±0.07 mm，Yellowish-white, with mouth hooks, flat cone-shaped protruding embryo	Mucosa of nasal cavity and throat	Every year from February to April
Third stage	Body length 23.17±0.04 mm, width 7.71±0.02 mm，the aspect ratio is about 3:1, the cephalic segment has short antennae, cone-shaped protrusions, and stomata on the valve plate	Throat	Every year from April to September

Partially collected sample photos

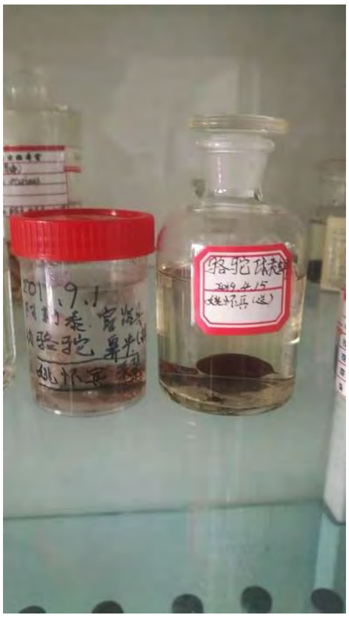

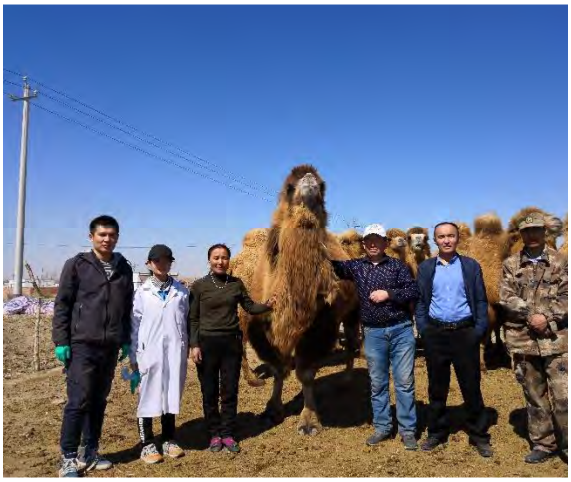

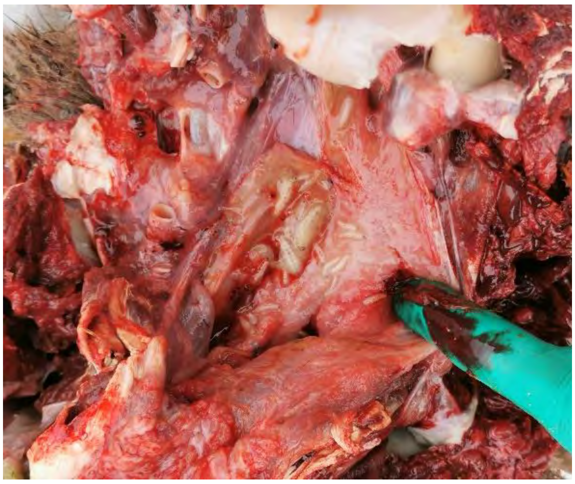

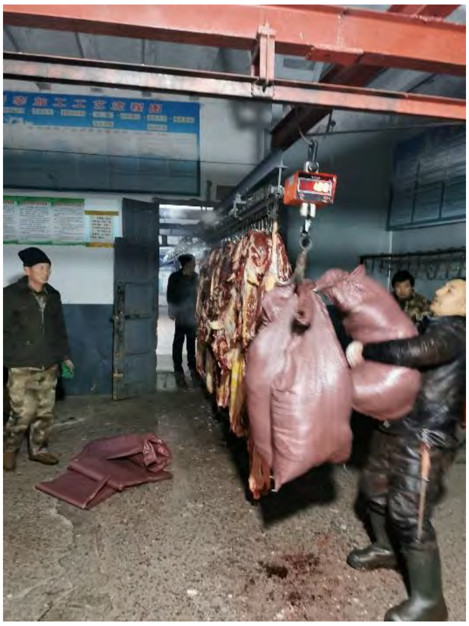

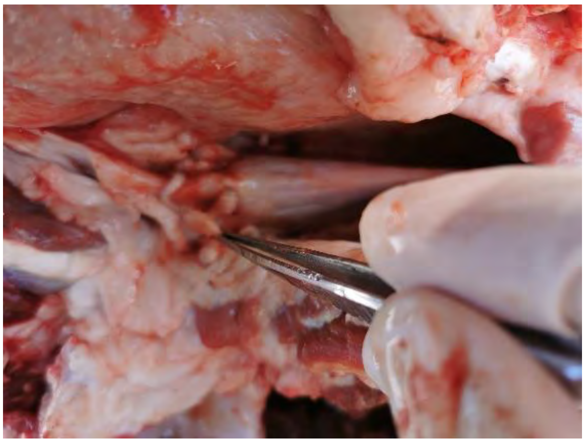

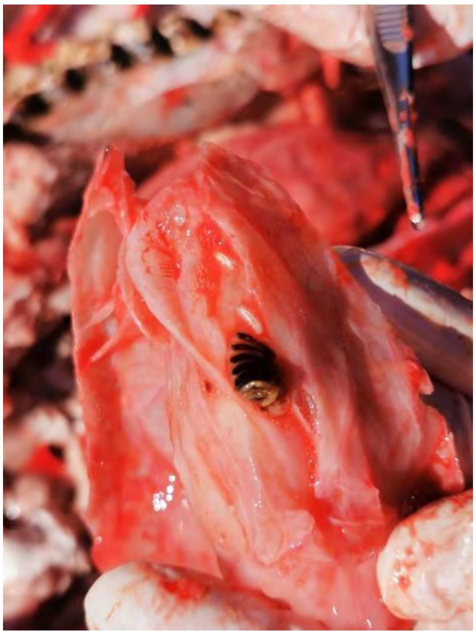

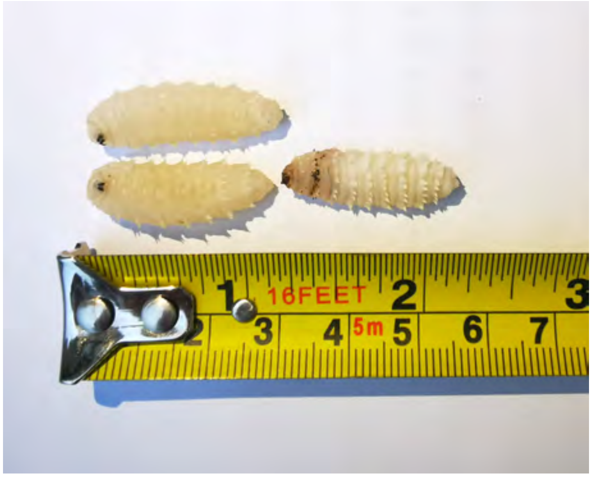

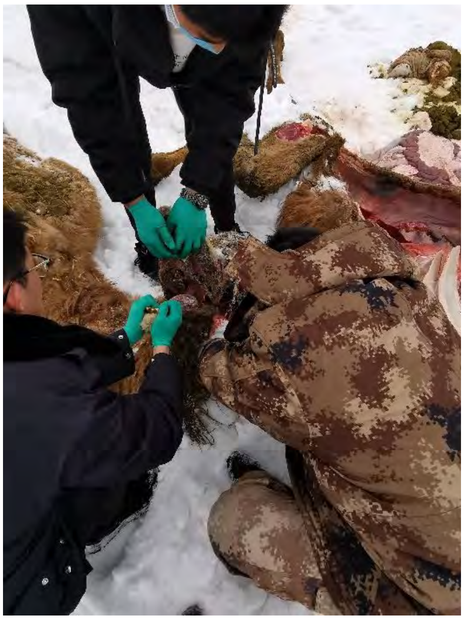
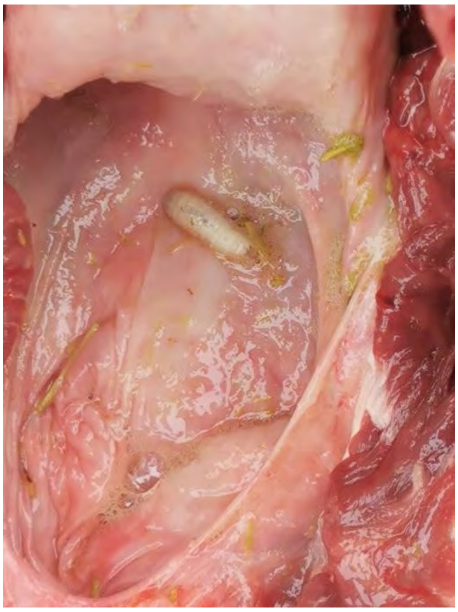
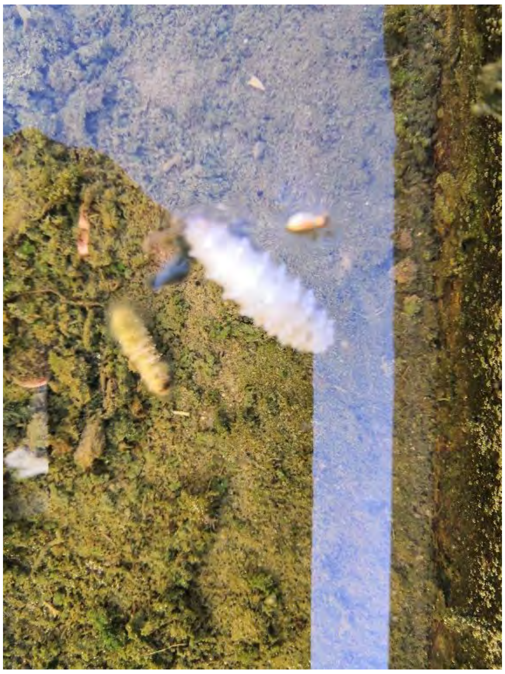
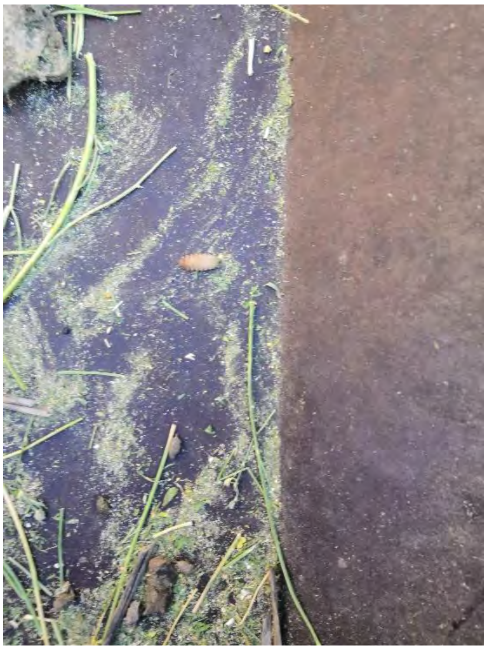
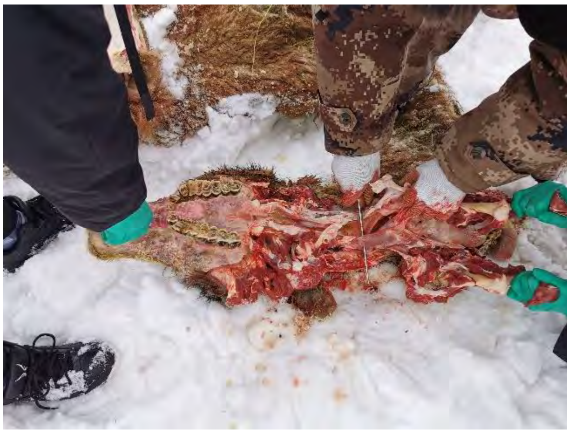
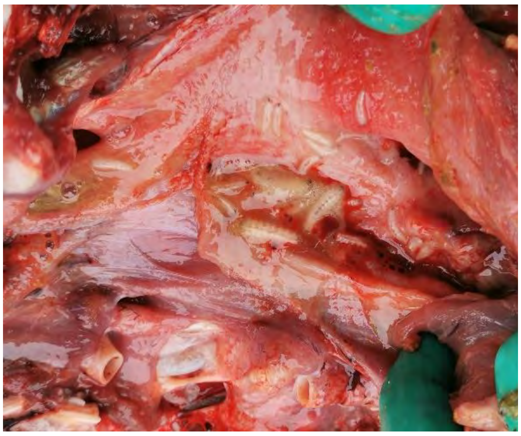

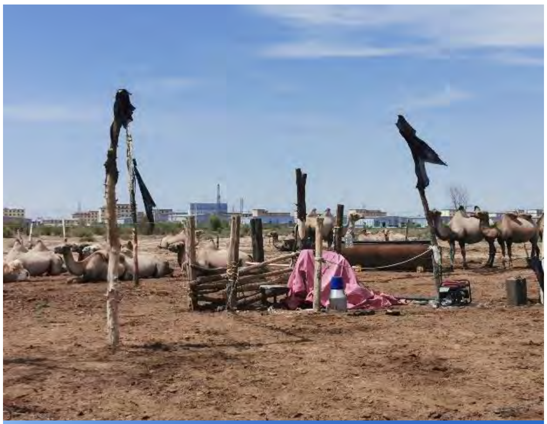
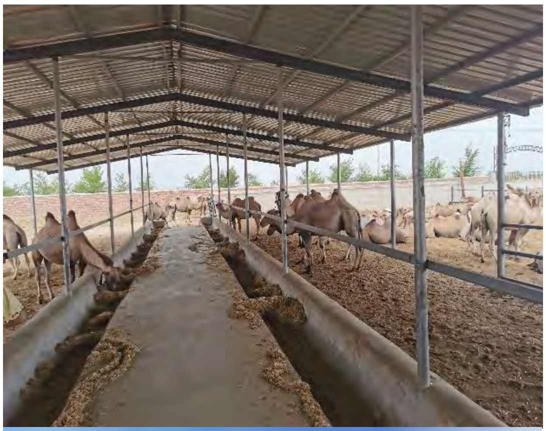
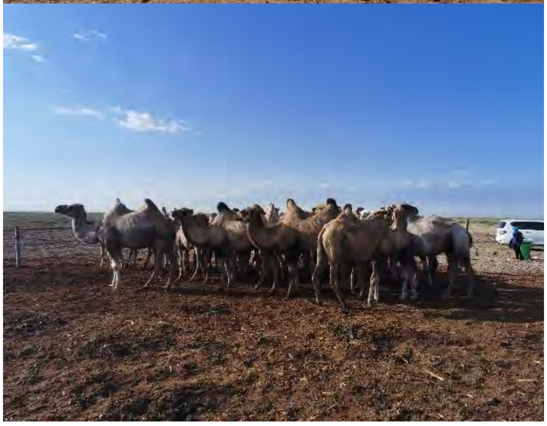
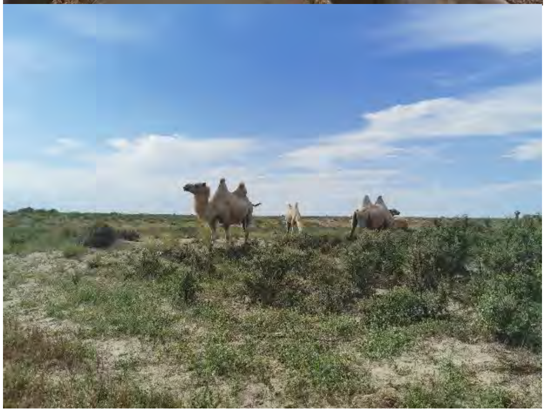
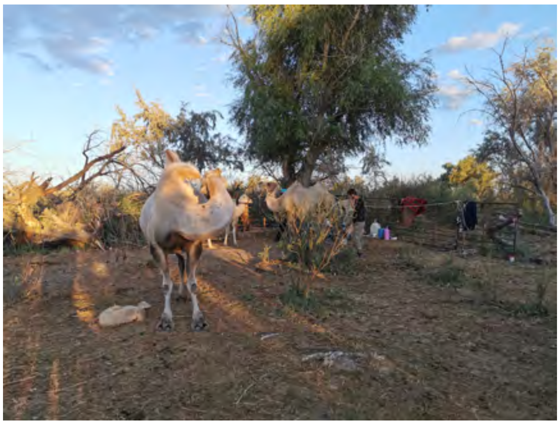
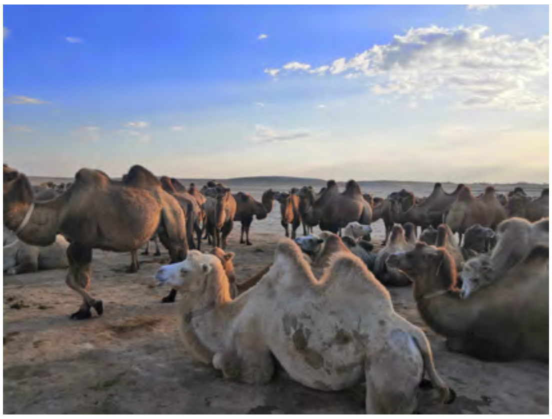
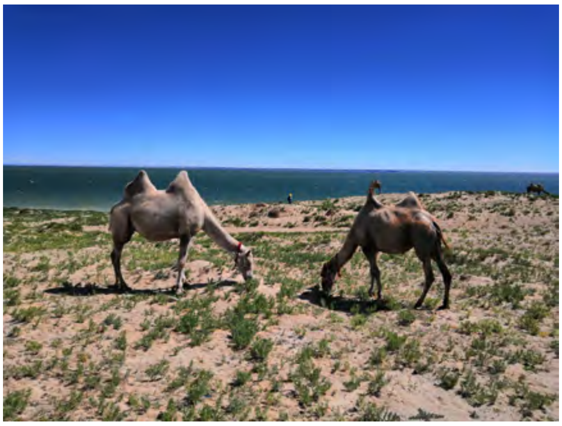
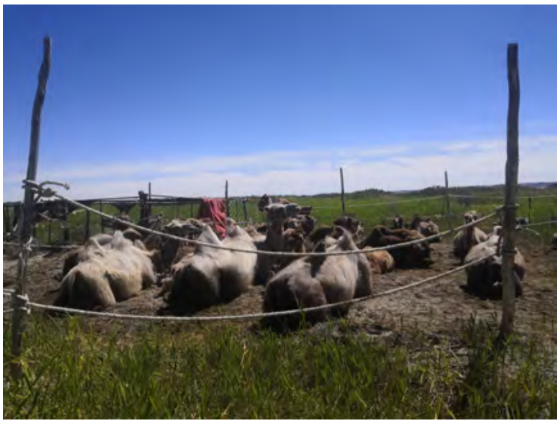
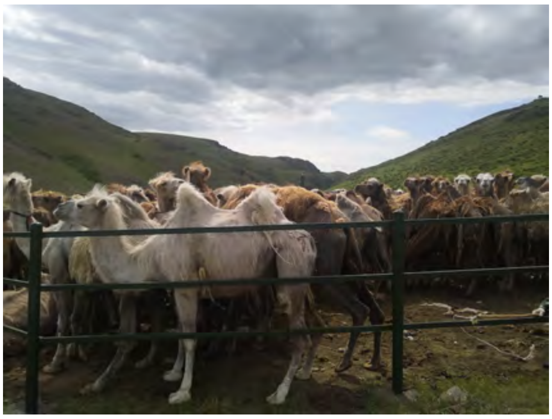
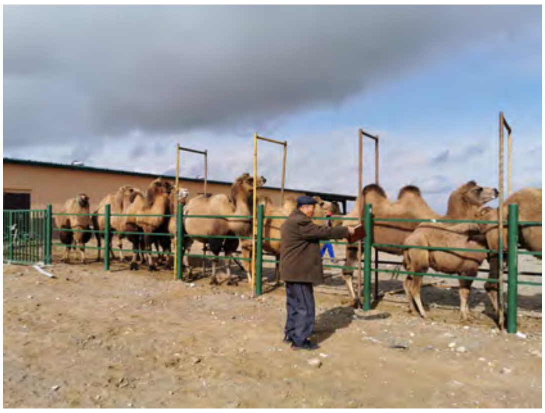

Cephalopina titillator isolate Larva01 cytochrome c oxidase subunit I (COX1) gene, partial cds; mitochondrial
GenBank: MZ152916.1

```
LOCUS       MZ152916               1275 bp    DNA     linear   INV 15-MAY-2021
DEFINITION  Cephalopina titillator isolate Larva01 cytochrome c oxidase subunit
            I (COX1) gene, partial cds; mitochondrial.
ACCESSION   MZ152916
VERSION     MZ152916.1
KEYWORDS    .
SOURCE      mitochondrion Cephalopina titillator
  ORGANISM  Cephalopina titillator
            Eukaryota; Metazoa; Ecdysozoa; Arthropoda; Hexapoda; Insecta;
            Pterygota; Neoptera; Endopterygota; Diptera; Brachycera;
            Muscomorpha; Oestroidea; Oestridae; Hypodermatinae; Cephalopina.
REFERENCE   1  (bases 1 to 1275)
  AUTHORS   Yao,H.B., Liu,M.L., Ma,W.P., Song,R.Q., Ma,Q., Li,L., Wu,Z.Y.,
            Ma,Y.J., Yue,H.T., Chen,G.L., Chen,B.J. and Yang,J.
  TITLE     Prevalence and pathology of Cephalopina titillator in camels
            (Camelus bactrianus) in Xinjiang, China
  JOURNAL   Unpublished
REFERENCE   2  (bases 1 to 1275)
  AUTHORS   Yao,H.B., Yang,J., Yue,H.T., Liu,M.L., Ma,W.P., Song,R.Q., Ma,Q.,
            Li,L., Ma,Y.J., Wu,Z.Y., Chen,G.L. and Chen,B.J.
  TITLE     Direct Submission
  JOURNAL   Submitted (10-MAY-2021) College of Life Sciences and Technology,
            Xinjiang University, 666 Shengli Road Tianshan District, Urumqi,
            Xinjiang 830046, China
COMMENT     ##Assembly-Data-START##
            Sequencing Technology :: Sanger dideoxy sequencing
            ##Assembly-Data-END##
FEATURES             Location/Qualifiers
     source          1..1275
                     /organism="Cephalopina titillator"
                     /organelle="mitochondrion"
                     /mol_type="genomic DNA"
                     /isolate="Larva01"
                     /isolation_source="Nasal passages and pharynx of the camel
                     heads"
                     /host="Camelus bactrianus"
                     /specimen_voucher="Cti"
                     /db_xref="taxon:2707115"
                     /country="China"
                     /collection_date="Mar-2021"
                     /PCR_primers="fwd_seq: atttataatgtaattgtcaccgcac, rev_seq:
                     ttggtaattcggcatatctgtgt"
                     /note="breed: Junggar Bactrian Camels"
     gene            <1..>1275
                     /gene="COX1"
     CDS             <1..>1275
                     /gene="COX1"
                     /codon_start=2
                     /transl_table=5
                     /product="cytochrome c oxidase subunit I"
                     /protein_id="QUX45037.1"
                     /translation="FMVMPIMIGGFGNWLVPLMLGAPDMAFPRMNNMSFWLLPPALTL
                     LLTSSMVESGAGTGWTVYPPLSSNIAHSGASVDLAIFSLHLAGISSILGAVNFITTII
                     NMRSIGMTLDRTPLFVWSVMITAILLLLSLPVLAGAITMLLTDRNLNTSFFDPAGGGD
                     PILYQHLFWFFGHPEVYILILPGFGMISHIISQESGKKETFGALGMIYAMLAIGLLGF
```

IVWAHHMFTVGMDIDTRAYFTSATMIIAVPTGIKIFSWLATLYGTQLNWSPAMLWSLG
FVFLFTVGGLTGVVLANSSVDIMLHDTYYVVAHFHYVLSMGAVFAIMGGFIHWFPLFT
GLTFNKMLLQTQFIVMFMGVNLTFFPQHFLGLSGMPRRYSDYPDAYTAWNVISSIGST
ISFLGILMFMYIIWESLTSQRKILFSNQLNSS"

ORIGIN

```
        1 ctttatagtt ataccaatta taattggagg atttggaaat tgactagtcc cactaatact
       61 aggggcccca gatatagcat tccctcgaat aaataatata agattctgac ttttacctcc
      121 cgctcttaca ctccttctaa caagaagaat agtagaaagc ggcgctggca ctggatgaac
      181 tgtttatcct cctctttcat caaatattgc ccacagagga gcctccgtag atttagcaat
      241 tttctcactt catttagctg gaatttcatc cattttagga gcagttaatt ttattacaac
      301 aatcatcaat atacgatcta ttggaataac tttagatcga acacccttat ttgtatgatc
      361 tgtaataatt acagcaattc ttttacttct atctctacca gttttagcag gagccattac
      421 aatactatta accgaccgga atttaaacac atcatttttt gaccctgcag gagggggaga
      481 ccccattctt tatcaacact tattttgatt ttttggacac cccgaagttt acattttaat
      541 tctccccgga ttcggaataa tttcccatat tattagacaa gaatcaggaa aaaaggaaac
      601 ctttggagct ctaggaataa tctacgccat attagccatt ggactgttag gattcattgt
      661 ttgagcccac cacatattta cagttggaat agatattgat acccgagctt actttacttc
      721 tgctacaata atcattgccg ttcctacggg aattaaaatt tttagctgac tagccacact
      781 atatggcacc caactaaact gatctcctgc catattatga tcactaggat tcgtatttct
      841 atttacagtg ggcggattaa caggagtagt actagctaat tcatccgtag acattatatt
      901 acatgataca tattatgttg tagcccactt ccattacgtt ttatcaatag gagcagtttt
      961 cgctatcata ggaggattta ttcactgatt ccccctattt actggattaa catttaataa
     1021 aatacttctt caaacacaat ttattgtaat atttatagga gtaaatttaa cattcttccc
     1081 ccaacacttc cttggactct ccggcatacc ccgccgatat tcagattacc cagatgcata
     1141 tactgcttga aatgtaatct cttcaatcgg atcaacaatt tcatttctag gaattttaat
     1201 atttatatat attatctgag aaagattaac atctcaacga aaaattttat tttcaaatca
     1261 attaaactca tcaat
//
```

Cephalopina titillator cytochrome b (CytB) gene, partial cds; mitochondrial
GenBank: MZ189361.1
LOCUS MZ189361 809 bp DNA linear INV 20-DEC-2021
DEFINITION Cephalopina titillator cytochrome b (CytB) gene, partial cds;
mitochondrial.
ACCESSION MZ189361
VERSION MZ189361.1
KEYWORDS .
SOURCE mitochondrion Cephalopina titillator
ORGANISM Cephalopina titillator
Eukaryota; Metazoa; Ecdysozoa; Arthropoda; Hexapoda; Insecta;
Pterygota; Neoptera; Endopterygota; Diptera; Brachycera;
Muscomorpha; Oestroidea; Oestridae; Hypodermatinae; Cephalopina.
REFERENCE 1 (bases 1 to 809)
AUTHORS Yao,H.B., Liu,M.L., Ma,W.P., Song,R.Q., Ma,Q., Li,L., Wu,Z.Y.,
Ma,Y.J., Yue,H.T., Chen,G.L., Chen,B.J. and Yang,J.
TITLE Prevalence and pathology of Cephalopina titillator in camels
(Camelus bactrianus) in Xinjiang, China
JOURNAL Unpublished
REFERENCE 2 (bases 1 to 809)
AUTHORS Yao,H.B., Yang,J., Yue,H.T., Liu,M.L., Ma,W.P., Song,R.Q., Ma,Q.,
Li,L., Ma,Y.J., Wu,Z.Y., Chen,G.L. and Chen,B.J.
TITLE Direct Submission
JOURNAL Submitted (11-MAY-2021) College of Life Sciences and Technology,
Xinjiang University, 666 Shengli Road, Tianshan District, Urumqi,
Xinjiang 830046, China
COMMENT ##Assembly-Data-START##
Sequencing Technology :: Sanger dideoxy sequencing
##Assembly-Data-END##
FEATURES Location/Qualifiers
source 1..809
/organism="Cephalopina titillator"
/organelle="mitochondrion"
/mol_type="genomic DNA"
/isolate="Larva01"
/isolation_source="nasal passages and pharynx of the camel
heads"
/host="Camelus bactrianus breed Junggar Bactrian"
/specimen_voucher="Cti"
/db_xref="taxon:2707115"
/dev_stage="larva"
/country="China"
/collection_date="Mar-2021"
/PCR_primers="fwd_seq: gattattcctcgccatacattacac, rev_seq:
gattattcctcgccatacattacac"
gene <1..>809
/gene="CytB"
CDS <1..>809
/gene="CytB"
/codon_start=3
/transl_table=5
/product="cytochrome b"
/protein_id="UGY70893.1"
/translation="HICRDVNYGWILRTLHANGASFFFICIYFHIGRGIYYNSYTFTP
TWMIGVIILFLLMATAFMGYVLPWGQMSFWGATVITNLLSAIPYLGTTLVQWIWGGFA
VDNATLTRFFTFHFILPFIVLATTLIHILFLHETGSNNPLGINSNVNKIPFHPYFTYK
DIVGFSMMLMALILLTLMNPYLLGDPDNFIPANPLVTPIHIQPEWYFLFAYAILRSIP

NKLGGVIALIMSIAILMILPFYHMSKFRGIQFYPINEILFWTMIITIILLT"

ORIGIN

1 accatatttg ccgggatgtt aattatggat gaattcttcg aacccttcat gccaatggag
61 catcattttt tttcatctgc atttactttc acattggtcg aggaatttat tataactcat
121 atacctttac ccccacatga ataattggag taattattct attcctatta atagcaacag
181 catttatagg atatgtactc ccatgaggac aaatatcatt ttgaggcgcc acagtaatta
241 ctaatcttct atcagcaatc ccatacttag ggactacttt agtacaatga atttgaggag
301 gcttcgcagt agacaatgcc actcttacac gattctttac attccacttc attctaccct
361 ttattgttct tgccacaact ttaattcata tcttatttct acacgaaaca ggatctaata
421 accccctagg aatcaactca aacgtaaata aaatcccctt ccacccctac tttacctaca
481 aagacatcgt aggattttca ataatattaa tagcattgat tctattaaca ctaataaatc
541 cctacctact aggagaccca gacaacttca tcccagcaaa ccctctagta accccccattc
601 atatccaacc tgaatgatac tttttattcg cttacgcaat tttacgctca atccccaata
661 aattaggagg agtaattgca ctaattatat caattgcaat tctaatgatt ctcccatttt
721 accatataag aaaatttcga ggaattcaat tctatcctat taatgaaatt ctattttgaa
781 caataattat tacaattatc ctacttaca

//

Cephalopina titillator isolate Larva01 cytochrome c oxidase subunit I (COX1) gene, partial cds; mitochondrial
GenBank: MZ209004.1
LOCUS MZ209004 529 bp DNA linear INV 22-MAY-2021
DEFINITION Cephalopina titillator isolate Larva01 cytochrome c oxidase subunit
I (COX1) gene, partial cds; mitochondrial.
ACCESSION MZ209004
VERSION MZ209004.1
KEYWORDS .
SOURCE mitochondrion Cephalopina titillator
ORGANISM Cephalopina titillator
Eukaryota; Metazoa; Ecdysozoa; Arthropoda; Hexapoda; Insecta;
Pterygota; Neoptera; Endopterygota; Diptera; Brachycera;
Muscomorpha; Oestroidea; Oestridae; Hypodermatinae; Cephalopina.
REFERENCE 1 (bases 1 to 529)
AUTHORS Yao,H.B., Liu,M.L., Ma,W.P., Song,R.Q., Ma,Q., Li,L., Wu,Z.Y.,
Ma,Y.J., Yue,H.T., Chen,G.L., Chen,B.J. and Yang,J.
TITLE Prevalence and pathology of Cephalopina titillator in camels
(Camelus bactrianus) in Xinjiang, China
JOURNAL Unpublished
REFERENCE 2 (bases 1 to 529)
AUTHORS Yao,H.B., Yang,J., Yue,H.T., Liu,M.L., Ma,W.P., Song,R.Q., Ma,Q.,
Li,L., Ma,Y.J., Wu,Z.Y., Chen,G.L. and Chen,B.J.
TITLE Direct Submission
JOURNAL Submitted (17-MAY-2021) College of Life Sciences and Technology,
Xinjiang University, 666 Shengli Road Tianshan District, Urumqi,
Xinjiang 830046, China
COMMENT ##Assembly-Data-START##
Sequencing Technology :: Sanger dideoxy sequencing
##Assembly-Data-END##
FEATURES Location/Qualifiers
source 1..529
/organism="Cephalopina titillator"
/organelle="mitochondrion"
/mol_type="genomic DNA"
/isolate="Larva01"
/isolation_source="Nasal passages and pharynx of the camel
heads"
/host="Camelus bactrianus"
/db_xref="taxon:2707115"
/country="China: Xinjiang"
/collection_date="Mar-2021"
/identified_by="Electron micrographs and gene sequence"
/PCR_primers="fwd_seq: attaattcgaatagagctaggacac, rev_seq:
aatgatgtgtttaaattccggtc"
gene <1..>529
/gene="COX1"
CDS <1..>529
/gene="COX1"
/codon_start=2
/transl_table=5
/product="cytochrome c oxidase subunit I"
/protein_id="QVJ07658.1"
/translation="ELGHPGTLIGNDQIYNVIVTTHAFIMIFFMVMPIMIGGFGNWLV
PLMLGAPDMAFPRMNNMSFWLLPPALTLLLTSSMVESGAGTGWTVYPPLSSNIAHSGA
SVDLAIFSLHLAGISSILGAVNFITTIINMRSIGMTLDRTPLFVWSVMITAILLLLSL
PVLAGAITMLLTDRNL"
ORIGIN

1 agagctagga caccccggaa cgctcattgg aaatgatcaa atttataatg taattgtcac
61 cacacatgct ttcatcataa ttttctttat agttatacca attataattg gaggatttgg
121 aaattgacta gtcccactaa tactaggggc cccagatata gcattccctc gaataaataa
181 tataagattc tgacttttac ctcccgctct tacactcctt ctaacaagaa gaatagtaga
241 aagcggcgct ggcactggat gaactgttta tcctcctctt tcatcaaata ttgcccacag
301 aggagcctcc gtagatttag caattttctc acttcattta gctggaattt catccatttt
361 aggagcagtt aattttatta caacaatcat caatatacga tctattggaa taactttaga
421 tcgaacaccc ttatttgtat gatctgtaat aattacagca attcttttac ttctatctct
481 accagtttta gcaggagcca ttacaatact attaaccgac cggaattta
//
